# Phytochemical Profiling and Structure-Based Computational Characterization of *Marrubium vulgare* L. Compounds as Hsp90 Modulators

**DOI:** 10.3390/ijms262412150

**Published:** 2025-12-17

**Authors:** Ilham Zarguan, Hanane Abbou, Razana Zegrari, Rihab Festali, Devan Buchanan, Abdelaziz Benjouad, Lamiae Belayachi

**Affiliations:** 1 Health Sciences Research Center, College of Health Sciences, International University of Rabat, Technopolis Parc, Rocade of Rabat-Salé, Sala-Al Jadida 11100, Morocco; abdelaziz.benjouad@uir.ac.ma (A.B.); lamiae.belayachi@uir.ac.ma (L.B.); 2 Georgia Cancer Center, Augusta University, Augusta, GA 30912, USA; 3 Laboratory of Drug Sciences (LRSM), Mohammed VI Faculty of Pharmacy, Mohammed VI University of Sciences and Health (UM6SS), Casablanca 82403, Morocco; habbou@um6ss.ma (H.A.); rzegrari@um6ss.ma (R.Z.); 4 Mohammed VI Center for Research and Innovation (CM6RI), Rabat 10112, Morocco; rfestali@um6ss.ma; 5 Research Laboratory of Microbiology, Infectious Diseases, Allergology and Pathogen Surveillance (LARMIAS), Morocco UM6SS, Mohammed VI Faculty of Medicine Casablanca, Mohammed VI University of Sciences and Health, Casablanca 82403, Morocco; 6 Department of Chemistry and Biochemistry, Augusta University, Augusta, GA 30912, USA; 7 Higher School of Biomedical Engineering, Health Sciences Research Center, College of Health Sciences, International University of Rabat, Technopolis Parc, Rocade of Rabat-Salé, Sala-Al Jadida 11100, Morocco

**Keywords:** *Marrubium vulgare*, phytochemicals, HSP90 heat-shock proteins, computational biology, liquid chromatography–mass spectrometry, antineoplastic agents

## Abstract

*Marrubium vulgare* L. is a medicinal plant widely used in traditional medicine, with emerging evidence of anticancer potential. This study investigated its bioactive compounds as inhibitors of Heat Shock Protein 90 alpha (Hsp90α), a molecular chaperone essential for oncogenic protein stability. Organic and aqueous extracts were profiled using high-performance liquid chromatography–mass spectrometry (HPLC–MS), revealing a diverse phytochemical composition. Identified compounds were screened against the full-length crystal structure of Hsp90α using a structure-based computational workflow that included extra-precision and domain-specific molecular docking, molecular dynamics (MD) simulations, and MM/GBSA binding free energy calculations. Pharmacokinetic and toxicity profiles were evaluated through ADMET predictions. This study elucidated the chemical composition of the plant and identified two hit compounds: Forsythoside B bound preferentially to the middle domain, potentially interfering with client protein interactions, and chlorogenic acid targeted the C-terminal domain, which regulates dimerization and allosteric activity. Both ligands displayed stable protein–ligand interactions during MD and favorable ADMET properties. These findings provide the first integrated chemical and computational prediction framework, suggesting that some *M. vulgare* metabolites may interact with Hsp90, highlighting its potential as a source of novel anticancer scaffolds and laying the groundwork for experimental validation and drug development.

## 1. Introduction

Medicinal plants have long served as a foundation for traditional medicine and the development of modern therapeutic agents [1,2]. Among these, *Marrubium vulgare* L. (*M. vulgare*), commonly known as white horehound, has been used extensively for its broad pharmacological properties, including antioxidants, anti-inflammatory, antimicrobial, and antihypertensive effects [3,4]. This perennial member of the Lamiaceae family is native to the Mediterranean basin and has been widely adopted in folk medicine across Europe, North Africa, and the Middle East [5]. In Morocco, where the plant is known as “merriwet” or “M’riwta,” it holds significant ethnopharmacological value and is traditionally prescribed for respiratory disorders, hypertension, diabetes, gastrointestinal issues, and more [5]. Ethnobotanical surveys have demonstrated its continued use in regions like Meknes-Tafilalet and Southeastern Morocco, especially in areas with limited access to modern healthcare [6,7].

The medicinal effects of *M. vulgare* are attributed to its rich phytochemical composition, which includes several major classes of bioactive compounds [6,8]. The plant is particularly notable for its content of labdane diterpenes, with marrubiin being the signature bitter compound responsible for many of its traditional uses [9,10]. Phenolic compounds, including rosmarinic acid and other caffeic acid derivatives, contribute significantly to its antioxidant properties [11]. The flavonoid profile includes luteolin, apigenin, and their various glycosides, which have been associated with anti-inflammatory and potential anticancer activities [12]. Additional constituents include tannins, saponins, and essential oil components that vary with geographic origin and extraction methods [13]. Other components such as apigenin, luteolin, and various phenolics also contribute to its reported antimicrobial and anticancer activities [9,11]. Analytical techniques, such as High-Performance Liquid Chromatography (HPLC), have enabled the detailed chemical profiling of *M. vulgare*, facilitating the identification of active secondary metabolites [11]. Previous studies have demonstrated that M. vulgare extracts exhibit significant antioxidant activity through free radical scavenging mechanisms, with activity correlating to total phenolic content [14]. Anti-inflammatory effects have been documented both in vitro, through the inhibition of inflammatory mediators, and in vivo using animal models of acute inflammation [15]. These bioactivities provide scientific support for the plant’s traditional uses and suggest potential applications in modern medicine [16].

Despite its extensive traditional use and rich phytochemical composition, the molecular targets underlying the pharmacological effects of *M. vulgare* remain insufficiently characterized. A particularly promising strategy for identifying novel anticancer compounds involves targeting Heat shock protein 90 (Hsp90), a molecular chaperone essential for maintaining protein homeostasis and cell survival [17]. Hsp90 is a highly conserved molecular chaperone that regulates the folding, stabilization, and activation of more than 300 client proteins implicated in critical cellular processes such as signal transduction, cell cycle control, and oncogenesis [18]. The protein functions as a homodimer with three distinct domains per protomer: the N-terminal ATP-binding domain, the middle domain involved in client protein and co-chaperone interactions, and the C-terminal domain responsible for dimerization and allosteric regulation [19]. In cancer cells, Hsp90 is often overexpressed and plays a key role in stabilizing mutated and oncogenic proteins, thereby sustaining malignant phenotypes [20]. This makes it an attractive therapeutic target [21]. Current Hsp90 inhibitors can be broadly classified into two categories based on their binding sites and mechanisms of action. N-terminal inhibitors, such as geldanamycin and radicicol, are ATP-competitive ligands that bind to the N-terminal ATP-binding pocket and block the ATP-driven conformational cycle essential for client protein maturation [22]. While potent, these inhibitors strongly activate the heat shock response (HSR) through HSF1, leading to upregulation of cytoprotective heat shock proteins that can limit therapeutic efficacy [23]. C-terminal inhibitors, exemplified by novobiocin and its derivatives, bind to the C-terminal domain and disrupt Hsp90 function through allosteric mechanisms, interfering with dimerization and co-chaperone interactions [24]. Importantly, C-terminal inhibitors typically produce less robust HSR activation, potentially offering improved therapeutic windows [25]. Therefore, despite intensive drug development efforts, especially against the N-terminal ATP-binding domain, no Hsp90 inhibitor has yet received FDA approval due to dose-limiting toxicities and the emergence of resistance [26]. These limitations have prompted increasing interest in alternative strategies, including selective inhibition of the middle and C-terminal domains, which mediate client protein binding and dimerization [27], as well as isoform-selective or allosteric modulators with improved therapeutic windows.

The reported bioactivity and structural diversity of M. vulgare metabolites suggest that this plant may harbor compounds capable of modulating Hsp90 through novel mechanisms, particularly targeting non-N-terminal domains to avoid the limitations associated with current inhibitors [28]. Building on its long-standing ethnopharmacological use and well-established safety profile, *M. vulgare* was therefore selected as a candidate source for novel Hsp90 modulators. In this study, a combined approach of phytochemical profiling and computational analyses was applied to identify and characterize plant-derived compounds with the potential to target Hsp90 beyond its N-terminal domain, thereby contributing to the development of innovative, selective anticancer strategies.

## 2. Results

### 2.1. Plant Extraction

Sequential solvent extraction of *M. vulgare* (Voucher ID: RAB114877) aerial parts using a polarity-gradient strategy resulted in six chemically distinct fractions. A total of 80g of plant powder was extracted in two Soxhlet cycles using solvents of increasing polarity. From the first extraction sequence (hexane → ethyl acetate → ethanol), the yields obtained were hexane: 4.725%, ethyl acetate: 4.675%, and ethanol: 2.125%. From the second sequence (hexane → dichloromethane → methanol), the yields were hexane: 4.725%, dichloromethane: 3.825%, and methanol: 4.675%. In addition, an aqueous extract was prepared by cold maceration from 80 g of dried plant material in distilled water (5% *w*/*v*), followed by filtration and freeze-drying.

### 2.2. Chemical Analysis of M. vulgare Extracts

High-performance liquid chromatography coupled with mass spectrometry (HPLC-MS) was employed to profile the chemical composition of all six *M. vulgare* extracts. Across the dataset, a total of 40 distinct secondary metabolites were identified, with molecular weights ranging from 164.16 g/mol to 888.8 g/mol. The ethanol extract was the most chemically diverse, yielding 38 compounds, including several phenylpropanoid glycosides, flavonoids, and diterpenes. The ethyl acetate extract contained 17 compounds, while the methanol and dichloromethane extracts each yielded 10 compounds. The aqueous extract revealed 9 polar metabolites, and the non-polar hexane fraction contained 8 hydrophobic constituents. Each extract showed a unique phytochemical fingerprint reflective of the solvent’s polarity. The ethanol and ethyl acetate fractions were rich in glycosylated flavonoids (e.g., luteolin 7-O-glucuronide, apigenin 7-O-glucuronide), phenolic acids (rosmarinic acid, chlorogenic acid), and iridoid glycosides (forsythoside B, verbascoside). The aqueous extract, while less diverse, included key antioxidant constituents such as p-coumaric acid, apigenin 7-acetate, and eugenol. Non-polar extracts like hexane and dichloromethane were dominated by lipophilic compounds including β-sitosterol, hexadecane, and stigmast-5-en-3-ol.

**Figure 1:**
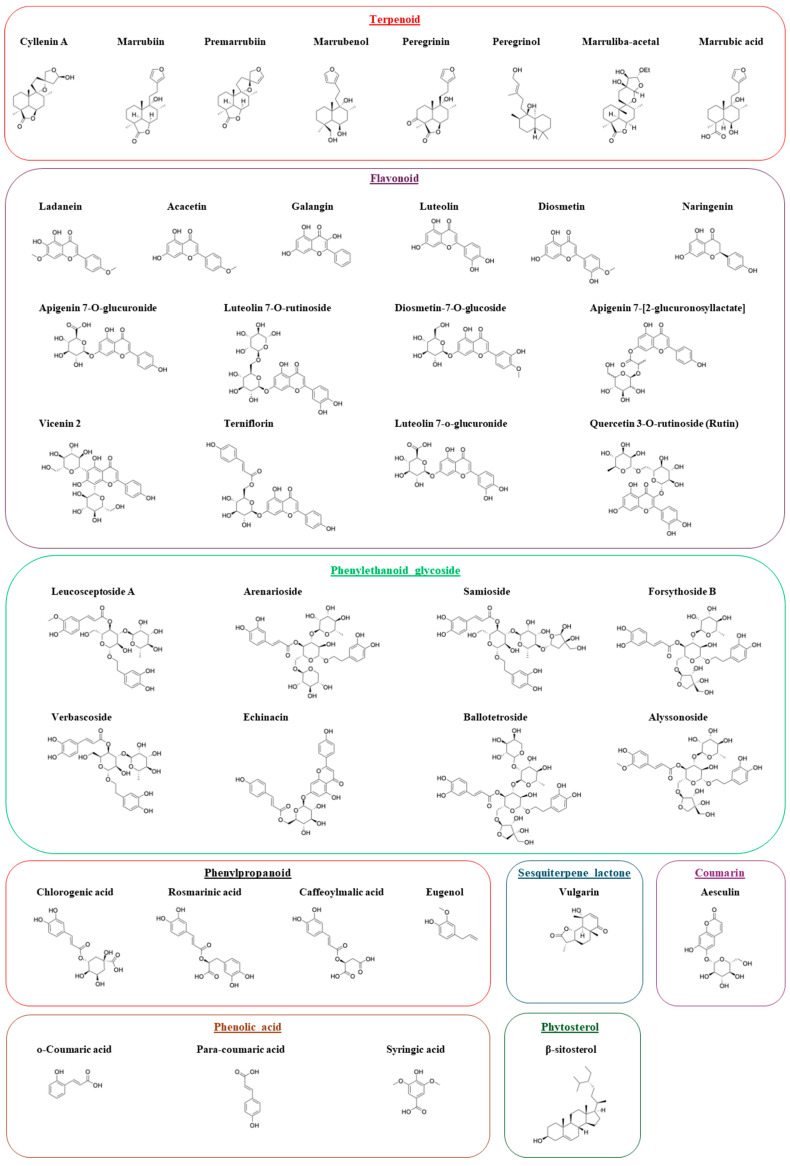
Chemical structures of the identified compounds from *M. vulgare* extracts identified using HPLC-MS and arranged based on chemical class. All chemical structures were constructed and reoriented using ChemDraw Professional (version 23.1.1).

The structural identities of all compounds were confirmed through MS fragmentation patterns and database matching. A comprehensive list of compounds identified in each extract, along with their molecular weights, is provided in Table 1, MS spectra of the identified compounds are provided in the Supplementary Table S1. The structures of representative compounds categorized by chemical class are shown in Figure 1.

### 2.3. Structures Preparation

**Figure 2:**
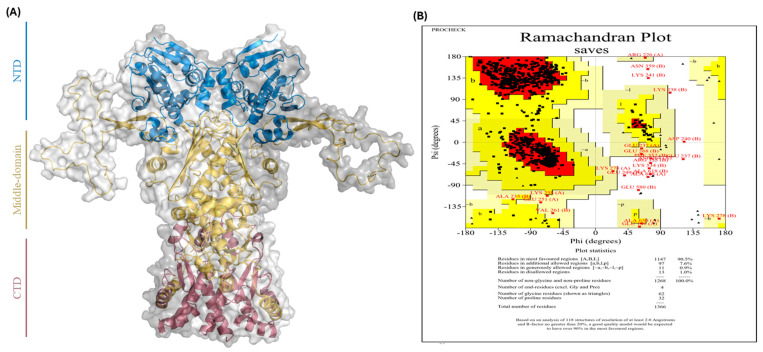
(**A**) 3D structure of full-length Hsp90α (PDB ID: 7KRJ) refined for molecular docking using SWISS-MODEL and visualized in PyMOL (PyMOL(TM) 3.1.6.1). (**B**) Ramachandran plot validation from PROCHECK. The distribution of backbone dihedral angles (φ and ψ) for all residues is shown. Red regions correspond to the most favored conformations, yellow regions to allowed conformations, pale yellow regions to generously allowed conformations, and white regions to disallowed conformations.

To investigate potential interactions with Hsp90α, identified *M. vulgare* compounds and known Hsp90 inhibitors were retrieved from PubChem and prepared using Open Babel and Schrödinger tools. The crystal structure of Hsp90α (PDB ID: 7KRJ) was refined by homology modeling via SWISS-MODEL to reconstruct missing N-terminal loops, and its quality was validated using PROCHECK, through Ramachandran plot analysis (Figure 2). The plot shows that less than 2% of the amino acids (24 residues) are outside the optimal and allowed areas. Since more than 90% of residues of the model fall within the optimal and allowed regions, the generated structure is considered reliable for further computational studies. The identified compounds, as well as known Hsp90 inhibitors for each binding site, were optimized using LigPrep, and the protein was pre-processed with the Protein Preparation Wizard. Molecular docking was then performed to explore their binding profiles.

### 2.4. Molecular Docking

Molecular docking simulations identified several phytochemicals with favorable binding affinities to non-ATP-binding regions of Hsp90α. The screening was specifically focused on the middle domain of chain A, the inter-chain middle-domain interface, and the C-terminal domain (CTD), deliberately excluding the N-terminal ATP-binding site to prioritize non-ATP-competitive inhibitors. Known Hsp90 inhibitors were used as a positive control for each domain, and only compounds with a better docking score were considered. Although, it is important to note that docking scores represent computational predictions of binding affinity and should be interpreted with caution, particularly when comparing structurally diverse compounds [29].

In the middle domain of chain A, Forsythoside B, Terniflorin, and Rosmarinic Acid exhibited the most favorable Glide scores of −6.84, −6.59, and −6.48, respectively. These compounds formed multiple hydrogen bonds and hydrophobic interactions with residues known to participate in client protein stabilization (Table 2).

In the inter-chain middle domain, Forsythoside B again ranked highest with a docking score of –7.96, significantly outperforming reference compounds such as Enniatin A. Several structurally related analogs from the phenylethanoid glycoside family (Figure 1 and Table 2), also exhibited high docking scores for the middle-domain, suggesting a conserved binding profile within this chemical class. Rosmarinic Acid emerged as the top-ranking compound from a different molecular class, with a score of −6.85 (Table 2).

Docking at the C-terminal domain, a region implicated in chaperone dimerization and allosteric regulation, highlighted Luteolin, Naringenin, and Chlorogenic Acid as the top binders, with Glide scores of −7.82, −7.50, and −7.33 kcal/mol, respectively. These affinities exceeded that of the C-terminal reference inhibitor Novobiocin (Table 2).

**Figure 3:**
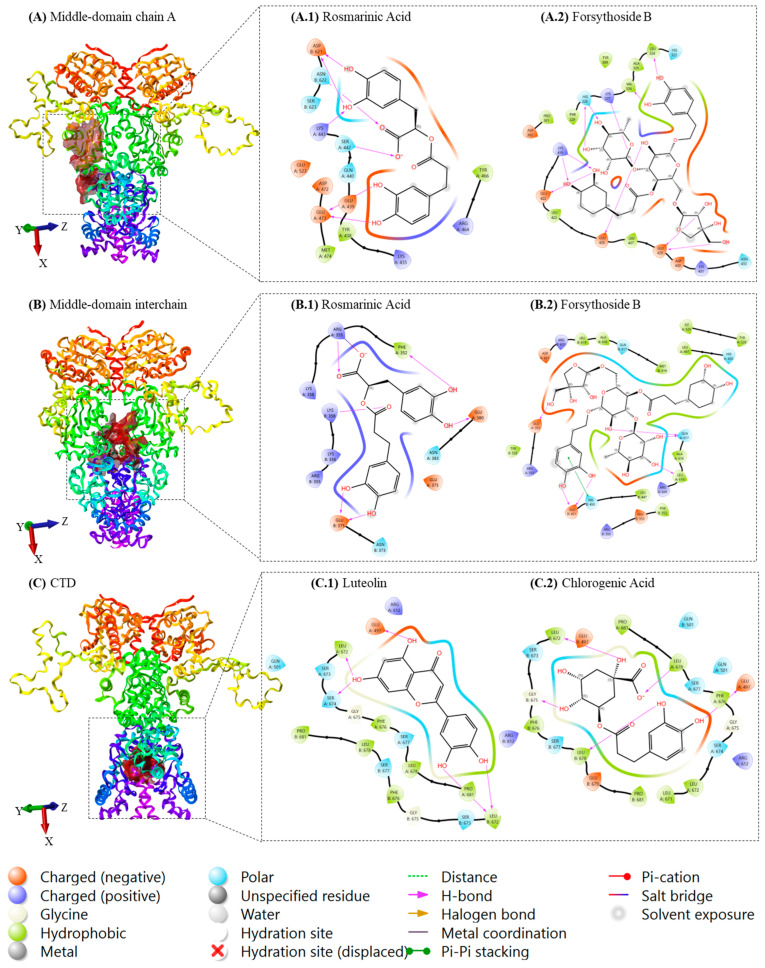
Binding poses of selected molecules for MD simulation. (**A.1**) Rosmarinic Acid and (**A.2**) Forsythoside B interaction with Hsp90α in (**A**) the middle-domain chain A. (**B.1**) Rosmarinic Acid and (**B.2**) Forsythoside B bound to (**B**) the interchain of the middle domain. (**C.1**) Luteolin and (**C.2**) Chlorogenic Acid bound to (**C**) the CTD. Three-dimensional structures of Hsp90α and Ligand–protein interactions were visualized and analyzed using Maestro.

Based on their superior docking scores and diverse chemotypes and acknowledging that direct comparison of docking energies between structurally distinct molecules should be interpreted as hypotheses of interaction rather than definitive affinity rankings, given that docking scores do not necessarily correlate with experimental binding free energies [30], a subset of compounds was selected for further analysis. Additionally, because the C-terminal domain of Hsp90 is highly dynamic, displays substantial flexibility, and lacks well-defined binding pockets, accurate computational prediction at this site remains challenging and requires validation through more advanced simulations or experimental methods [31]. Therefore, to further assess the stability and interaction dynamics of the top-scoring compounds, Forsythoside B and Rosmarinic Acid were chosen for molecular dynamics (MD) simulations at both the interchain interface and chain A of the middle domain, while Luteolin and Chlorogenic Acid were selected for MD simulations at the CTD (Figure 3).

### 2.5. Molecular Dynamics (MD) Simulations

MD simulation is a powerful computational tool for studying the movements of complex macromolecular systems, including biomolecules. It enhances molecular docking models by considering the flexibility of proteins and ligands, leading to a more accurate representation of the binding process and the identification of more effective drug candidates [32]. An important aspect of MD simulations is the analysis of the structural fluctuations of the macromolecule. It can be used to identify key conformational changes that occur upon ligand binding and subsequently provide insights into the molecular basis of ligand-target interactions [33]. The MD simulations were conducted to further investigate the stability and binding behavior of the top-scoring compounds identified from docking studies. Four compounds were selected for 100 ns MD simulations based on: (1) top docking scores from each domain, (2) structural diversity to represent different chemical classes, (3) preliminary drug-likeness assessment, and (4) literature precedent for bioactivity. The 100 ns simulation duration was chosen based on preliminary convergence analysis showing RMSD stabilization within this timeframe for most protein-ligand complexes [34]. The selected ligands included Forsythoside B and Rosmarinic Acid for the middle domain in both intra-chain and interchain sites, as well as Luteolin and Chlorogenic Acid for the CTD. These ligands were simulated in complex with Hsp90α to assess the dynamic nature of their interactions and their impact on protein flexibility over time. The simulation results indicated a stabilizing effect on the protein upon ligand binding, as compared to the unbound Hsp90α homodimer (Table 3). Additional validation metrics were analyzed, including hydrogen bond occupancy over time and ligand root mean square fluctuation (RMSF). These analyses revealed that forsythoside B maintained stable binding throughout the simulation, while luteolin showed some conformational instability in the C-terminal domain, consistent with the challenging nature of C-terminal binding predictions [35].

All the RMSD values have lower averages than Hsp90 (avg = 6.56 Å). More specifically, Forsythoside B bound to the middle domain of the protein’s Chain A showed the lowest RMSD values (avg = 5.25 Å). Still, when bound to the interchain of the middle domain, it showed less prominent stabilization (avg = 6.47 Å), but this value is still lower than the avg of Hsp90 alone. Interestingly, Rosmarinic Acid did not replicate the effect of Forsythoside B, as it managed to stabilize Hsp90 when it was bound to its Chain A middle domain, but failed to stabilize it when it was bound to the pocket between the middle domains of chain A and B of the dimer. Its MD simulation results in this domain were eliminated because the ligand continued to interact with multiple residues in various regions throughout the 100 ns simulation (Supplementary Table S3).

**Figure 4:**
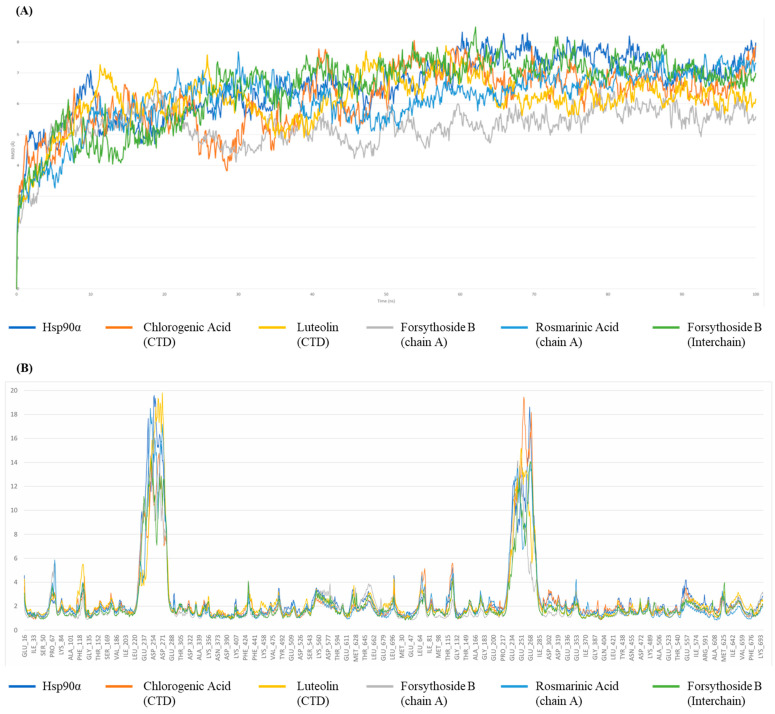
MD simulation analysis using Desmond. (**A**) RMSD plots of Hsp90α alone vs. protein–ligand complexes over 100 ns showing structural stability. (**B**) RMSF plots show residue-level flexibility during the simulation.

As for the C-terminal domain, both Chlorogenic acid and Luteolin exhibited similar patterns, with RMSD values lower than the unbound Hsp90 (avg = 6.16 Å and avg = 6.11 Å) (Figure 4). The RMSF values of all the systems follow the same trend as the RMSD values, as the Hsp90 protein has the highest RMSF values when it is unbound (avg = 2.65 Å), while the binding of Forsythoside B to the chain A middle domain lowers the average value down to 2.18 Å.

This correlation between RMSD and RMSF values is to be expected, as the presence of a ligand and its engagement in interactions with protein residues, especially when these interactions are stable over time, lowers the fluctuations of these specific residues and thus contributes to the limitation of the overall movement of the protein structure. On the other hand, Forsythoside B bound to the interchain binding pocket induces the second most noticeable effect on the RMSF values (avg = 2.25 Å). The remaining systems keep the same trend going, where the avg RMSF is lower than Hsp90 with no ligand.

A focus on the PLI gives an in-depth insight into the mechanism of action of each ligand and in each domain. Starting with Rosmarinic Acid in the middle domain of chain A, a considerable number of amino acids maintained a strong and consistent interaction with the ligand, including TYR438, GLU439, TYR466, GLU473, MET474, and LYS499. It is worth mentioning that periodically, the ligand would interact with additional proteins, but the aforementioned ones are those that could maintain stable and continuous interaction throughout the simulation time. The total contact number fluctuated between 1 and 15, with the most prominent values sitting at around 5 and 10 contacts per 0.1 ns. Upon close inspection of the RMSF values of these residues, a clear decline in all their individual RMSF was noticed, due to their constant engagement with stabilizing interactions with Rosmarinic Acid. For Forsythoside B in the middle domain of chain A, an interesting case arises, because the ligand interacts very tightly with ASP302, Val326, HIS328, GLU426, GLU429, and ASP430 up until the first 30 ns of the simulation, where it detached from the majority of the amino acids, and kept its interaction with GLU429 and ASP430, while initiating a new set of interactions with different amino acids, such as ASP479, THR482, ARG483, LYS485, GLU486, and GLU537. Coincidentally, the average number of interactions per ps dropped from 12 bonds within the first 30 ns to less than 6 for the remaining simulation time. We thus hypothesize that Forsythoside B in this domain has induced the most stabilizing effect because it targeted essential amino acids that are originally supposed to be flexible, rather than the number of bonds it formed with said residues. Forsythoside B interacts with the protein via a second region, which is the binding pocket between the middle domain of the two chains forming the dimer. The ligand has shown exceptional stability of a multitude of interactions, ranging from 10 to 20 interactions for the majority of the 100 ns simulation, with residues including PHE352, GLU353, ARG355, GLU451, GLN617, ALA618, and LEU619, on both chain A and B of the dimer, a few other residues in chain A such as GLU380, and on chain B such as TYR528, and HIS450.

On the CTD, Chlorogenic acid shows several interactions per 0.1 ns, fluctuating mostly between 10 and 15 bonds, with amino acids from both chains, specifically, GLU497, GLN501, ASN609, ARG612, SER677, and LEU678. On chain A, we find LYS615, SER674, and on chain B, GLY675, PHE676. All these amino acids display stable patterns of interaction with Chlorogenic Acid. As for Luteolin, the interaction profile appeared less prominent, due to the limited number of amino acids interacting with the ligand during the simulation time. The lasting interactions involve GLU497, ALA506, LEU672, and SER673 in chain A, and only Glu497 on chain B, but with an apparent weakening of the interaction by the end of the simulation time.

Based on the RMSD, RMSF, and PLIs, Forsythoside B at the interchain binding pocket and Chlorogenic acid at CTD were selected for MM/GBSA assessment.

### 2.6. Binding Free Energy Analysis

MM/GBSA binding free energy calculations provided a more rigorous assessment of protein-ligand interactions. The MM/GBSA analysis on the last 200 frames (20 ns) of the trajectory revealed that both molecules display good binding affinities with the protein (Table 4), with Forsythoside B showing a more favorable interaction profile. Energy decomposition analysis revealed that forsythoside B’s superior binding affinity (compared to reference inhibitor enniatin A) resulted from: (1) extensive hydrogen bonding networks (contributing ~40% of total binding energy), (2) favorable electrostatic interactions with charged residues, and (3) hydrophobic contacts with the interchain middle domain pocket [36]. The observation that rosmarinic acid stabilized the middle domain of chain A but not the interchain region appears to result from steric hindrance and residue-specific interactions unique to the interchain binding site, highlighting the importance of protein conformational context in ligand binding predictions [37].

### 2.7. ADMET Assessment

Finally, Chlorogenic acid and forsythoside B were identified as hit compounds based on their favorable binding profiles to Hsp90α observed through molecular docking and MD simulations and validated with MM/GBSA analysis. Following their selection, pharmacokinetic and toxicity predictions were carried out using SwissADME and ProTox 3.0.

According to ProTox 3.0, both compounds exhibited low acute oral toxicity, with a predicted LD_50_ of 5000 mg/kg and classification under toxicity class 5. Neither compound was predicted to be mutagenic or carcinogenic (Supplementary Table S4 for chlorogenic acid; Supplementary Table S5 for forsythoside B). However, both were flagged for potential immunotoxicity and nephrotoxicity, and forsythoside B was additionally predicted to be cardiotoxic.

SwissADME results indicated low gastrointestinal absorption and no blood–brain barrier (BBB) permeability for both compounds, consistent with their high topological polar surface areas (TPSA = 164.75 Å^2^ for chlorogenic acid and 304.21 Å^2^ for forsythoside B). Chlorogenic acid met most drug-likeness rules, with only one Lipinski violation and a bioavailability score of 0.11. In contrast, forsythoside B showed multiple violations of drug-likeness rules due to its large molecular weight (756.7 g/mol), high number of hydrogen bond donors/acceptors, and extensive polarity, with a slightly higher bioavailability score of 0.17 (Table 5). Neither compound was predicted to inhibit any of the major cytochrome P450 enzymes (Supplementary Table S6).

## 3. Discussion

*M. vulgare* is a widely used medicinal plant across North Africa, the Mediterranean, and Europe due to its reported antioxidant, anti-inflammatory, and anticancer activities [36]. It has a rich chemical composition and significant bioactive properties. The plant’s essential oil is primarily composed of E-caryophyllene (35.7%), germacrene D (25.2%), and bicyclogermacrene (10.6%) [37]. Additionally, the volatile fraction includes compounds such as 3-Thujanone, Eugenol, Topanol, Menthone, and Piperitone [38]. The plant’s bioactive compounds include a variety of polyphenols, flavonoids, phenolic acids, and tannins, with notable constituents like ferulic acid, catechin, quercetin, protocatechuic acid, rutin, and syringic acid [37]. *M. vulgare* also contains labdane diterpenes, with marrubiin being the major component, produced from premarrubiin under heating conditions [39]. Other identified compounds include alkaloids, sterols, steroids, terpenoids, saponins, catecholic tannins, and anthocyanins [40]. The plant exhibits a range of biological activities, including antioxidant, antimicrobial, anti-inflammatory, and antidiabetic effects [37,40]. These diverse phytochemicals and their associated biological activities underscore *M. vulgare*’s potential for therapeutic applications in pharmaceuticals and cosmetics. However, despite its widespread ethnomedicinal application, this plant remains underexplored from a molecular pharmacology perspective. To valorize both the phytochemical richness of *M. vulgare* and the ancient ethnopharmacological knowledge surrounding it, this study aimed to identify bioactive compounds from 5 organic and 1 aqueous extract and evaluate their potential against a clinically relevant target: Heat Shock Protein 90 (Hsp90), a key molecular chaperone involved in cancer progression and drug resistance [26,27]. In this study, we combined phytochemical screening, HPLC-MS profiling, and computational analyses to evaluate *M. vulgare* compounds as potential Hsp90 modulators, given Hsp90’s central role in stabilizing oncogenic client proteins and its relevance as a target in anticancer drug discovery. The rationale behind targeting Hsp90 with plant-derived compounds is supported by a growing body of literature, including recent works [41,42], which highlights the potential of natural products in modulating Hsp90 pathways in cancer models. This aligns with a broader shift in cancer therapeutics favoring selective modulation of protein networks over broad-spectrum inhibition, particularly when aiming to reduce side effects and improve tolerability.

Phytochemical analyses of *M. vulgare* revealed several bioactive molecules with potential anticancer activity, including flavonoids (luteolin, apigenin), phenylpropanoids (forsythoside B, rosmarinic acid), and phenolic acids (syringic, para-coumaric, chlorogenic) [43]. HPLC-MS profiling confirmed eight different chemical classes rich in flavonoids, phenolic acids, terpenoids, and phenylpropanoid glycosides. The ethanol and ethyl acetate extracts were abundant in glycosylated flavonoids such as luteolin and apigenin derivatives, phenolic acids like rosmarinic and chlorogenic acids, and iridoid glycosides including forsythoside B and verbascoside. These compound classes have been frequently associated with antioxidants, anti-inflammatory, and anticancer properties [44,45].

Understanding the distinct mechanisms of Hsp90 domain-specific inhibition is essential for rational drug design [39]. N-terminal inhibitors like geldanamycin and radicicol compete with ATP binding and generally induce client protein degradation and heat shock response activation [40]. In contrast, C-terminal allosteric modulators such as novobiocin disrupt protein dimerization and cochaperone interactions without triggering heat shock responses, potentially offering therapeutic advantages [41]. Our multi-domain docking strategy was designed to explore an alternative middle domain and C-terminal modulation approaches [42]. In our search for novel Hsp90 modulators, four phytochemicals were selected: Forsythoside B, Rosmarinic Acid, Luteolin, and Chlorogenic Acid based on their docking scores and structural attributes. Forsythoside B and rosmarinic acid showed favorable docking to the middle domain of Hsp90α, a region implicated in client and co-chaperone binding interactions [46]. Among natural–product Hsp90 modulators acting beyond the classical ATP–binding N–terminal pocket, examples such as Enniatin A (a cyclic hexadepsipeptide) [47] and Diptoindonesin G (dip G) [48] have recently emerged as middle–domain (MD) binders, capable of destabilizing client proteins without triggering the canonical heat-shock response (HSR). Enniatin A was shown to impair Hsp90 function in cancer cells, provoke client degradation and immunogenic cell death, while avoiding upregulation of the heat-shock proteins Hsp70, Hsp40 and Hsp27, which offer a major advantage over classical N–terminal inhibitors. Dip G binds the MD of Hsp90 with K(d) ≈ 0.13 µM and selectively degrades client oncoproteins (e.g., ERα) without HSR induction [48]. These modulators, although chemically diverse (peptidic for Enniatin A, small–molecule natural for dip G), illustrate that the Middle Domain of HSP90 is druggable by non–canonical scaffolds.

In this context, Forsythoside B represents a complementary yet distinct pharmacophoric strategy. Rather than relying on hydrophobic cyclic or rigid small–molecule cores, Forsythoside B is a relatively large, polyhydroxylated glycoside, featuring aromatic rings and sugar moieties that offer multiple hydrogen–bond donors/acceptors and high polarity. In the extra precision multi–domain docking and MD simulations, Forsythoside B persistently engaged the Middle Domain residues such as PHE352, GLU353, ARG355 and even inter–domain residue GLN617, and concomitantly reduces flexibility at ASP302, HIS328, and GLU429, suggesting stabilization of loops/helix elements and potential interference with inter–domain communication or client/co–chaperone binding. These features indicate that Forsythoside B might act via an allosteric mechanism distinct from the hydrophobic insertion or amphipathic cyclic binding seen with Enniatin A or dip G, but nonetheless able to perturb Hsp90 dynamics. Therefore, Forsythoside B expands the chemical and pharmacophoric diversity of Middle Domain–targeting Hsp90 ligands, underscoring the feasibility of “polar–scaffold” natural products as viable allosteric modulators of Hsp90. In contrast, rosmarinic acid had weaker and more localized interactions, suggesting lower efficacy as a modulator.

Luteolin and chlorogenic acid were both predicted to target the C-terminal domain (CTD) of Hsp90, a region essential for dimerization, co-chaperone association, and an increasingly attractive site for selective inhibition [46]. Importantly, Luteolin has already been experimentally reported as an Hsp90 modulator. Fu et al. demonstrated that Luteolin directly interacts with Hsp90, interferes with ATP binding, and disrupts the Hsp90–client protein complex, ultimately promoting degradation of client proteins such as STAT3 [49]. This prior evidence strongly supports the biological relevance of Luteolin as an Hsp90-active scaffold and provides additional validation for our computational pipeline, as our approach successfully identified a compound already known to modulate Hsp90 function. In the MD simulations, Luteolin maintained a stable interaction pattern within the CTD environment, consistent with its previously described capacity to engage Hsp90. Chlorogenic Acid, however, demonstrated an even more favorable interaction profile, forming persistent hydrogen bonds and hydrophobic contacts with key CTD residues including GLU497, GLN501, and ARG612 across both chains. Its RMSD and RMSF values (averaging 6.16 Å and below 2.0 Å, respectively) suggest moderate stabilization and selective engagement of the CTD compared with the unbound protein.

Chlorogenic acid has been reported to influence pathways involving Hsp90 client proteins, although it has not been characterized as a direct Hsp90 inhibitor, it has been shown to influence Hsp90-related signaling. A recent study found that it modulates transcription factor interactions with Hsp90: it weakens the SREBP2–Hsp90 complex and stabilizes PXR–Hsp90 binding, thereby altering nuclear translocation of these factors and regulating cholesterol homeostasis [50]. However, the only *M. vulgare* compound reported with direct evidence of Hsp90 inhibition is luteolin [51,52]. No peer-reviewed reports link forsythoside B or rosmarinic acid to direct Hsp90 modulation, although their predicted binding to the middle domain and known anti-inflammatory properties suggest potential for chaperone regulation.

Collectively, the combination of biochemical activity, drug-like properties, and structural compatibility led us to hypothesize that these compounds may act as Hsp90 modulators. Forsythoside B showed a strong modulatory effect with its ability to bind the middle domain and alter the protein function via rigidification of the amino acids and lowering its internal motion, thereby preventing its enzymatic activity and physically blocking the client protein recruitment pocket. Chlorogenic acid emerged as a moderate modulator; it binds specifically to the C-terminal domain and slightly lowers the protein flexibility, without affecting protein stability as strongly as forsythoside B. However, it is crucial to acknowledge that our findings are entirely computational in nature and require comprehensive experimental validation before any therapeutic claims can be substantiated. Each of these ligands could achieve a different biological response within the cell, depending on the degree to which we want to hinder the functionality of Hsp90. Further in vitro and in vivo studies would help clarify the difference each of these compounds would have on the protein and thus provide a clearer view of their therapeutic potential as Hsp90 modulators.

MM/GBSA free energy calculations confirmed forsythoside B as the most stable binder among the candidates [53], implying that interchain targeting compounds could serve as novel non-ATP-site modulators of Hsp90α [54,55]. ADMET analysis supported low acute oral toxicity for forsythoside B and chlorogenic acid (LD_50_ = 5000 mg/kg), no predicted mutagenicity or carcinogenicity, and absence of CYP450 inhibition. Forsythoside B, however, showed significant drug-likeness violations, particularly due to its large molecular weight (624 Da) and high polar surface area. These limitations do not preclude therapeutic potential but suggest the need for alternative development strategies [56]. Chlorogenic acid, on the other hand, demonstrated better drug-likeness properties and gastrointestinal absorption. Potential approaches to address pharmacokinetic limitations include: (1) structural modification to reduce molecular weight while maintaining key binding interactions, (2) prodrug strategies to improve absorption and bioavailability, (3) nanoparticle formulations to enhance delivery, and (4) consideration of these compounds as lead structures for optimization rather than direct drug candidates [57]. Natural product glycosides have historically served as valuable starting points for medicinal chemistry optimization [58,59].

## 4. Conclusions

In conclusion, this study characterized the phytochemical composition of *M. vulgare* across extracts of increasing polarity and aqueous extract and highlighted three key compounds with potential modulatory effects on Hsp90. Luteolin, a known Hsp90 inhibitor, reinforces the plant’s therapeutic relevance. Chlorogenic acid showed selective interaction with the C-terminal domain, indicating a modulatory but less disruptive role. Forsythoside B demonstrated promising binding to the middle domain, warranting further in vitro validation. These findings provide a computational prediction framework suggesting that some *M. vulgare* metabolites may interact with Hsp90 through novel mechanisms, warranting further biochemical and biological validation rather than supporting direct therapeutic claims. The study establishes a foundation for future experimental work that could validate these computational predictions and explore the therapeutic potential of *M. vulgare*-derived Hsp90 modulators. The integration of traditional medicinal knowledge with modern computational approaches demonstrates the continued value of natural products in drug discovery, while emphasizing the critical importance of experimental validation in translating computational predictions to therapeutic applications.

## 5. Materials and Methods

### 5.1. Plant Extraction

The leaves and stems of *M. vulgare* were collected in May 2025 from Rabat, Morocco (geographic coordinates: 34°0′47.7″ N 6°49.953′ W), authenticated at the RAB-Scientific Institute in Rabat, Morocco (voucher no. 114877), and air-dried separately in the shade under ventilated conditions at room temperature for five days until a stable weight was reached. The dried material was ground and sieved to obtain fine powder. Organic extraction was performed in two Soxhlet cycles (40 g/cycle) using solvents of increasing polarity: hexane → ethyl acetate → ethanol for the first cycle and hexane → dichloromethane → methanol for the second, with a mass-to-solvent ratio of 20/100 (g/mL) and each cycle lasting 5–12 h, using a Soxhlet apparatus (Agilent Technologies, anta Clara, CA, USA). Filtrates were concentrated under reduced pressure using a rotary evaporator (Büchi, Flawil, Switzerland), and crude extracts were stored at 4 °C. Aqueous extraction was performed by macerating 40g of powder in distilled water (20/100, *w*/*v*) for 24h at room temperature with stirring, followed by centrifugation, filtration, and concentration under reduced pressure at 60 °C. All extracts were stored at 4 °C in the dark until further use.

### 5.2. Chemical Analysis of M. vulgare Extracts

The chemical composition of *M. vulgare* Extracts was identified using high-performance liquid chromatography coupled with mass spectrometry (HPLC-MS) using an Ultimate 3000-Exactive plus system (Thermo Scientific, Waltham, MA, USA), at the National Centre for Scientific and Technical Research (CNRST) in Rabat. The analysis involved six extracts, which were diluted in MeOH (methanol) and analyzed along with the control (MeOH). The separation of compounds was achieved using a BDS HYPERSIL C18, dim. 150X4.6 mm particle size 5microm in polar mobile phase consisting of solvent A (water/0.5% formic acid) and solvent B (acetonitrile/0.5% formic acid). The gradient profile employed a constant flow rate of 0.5 mL/min, with a temperature of 30 °C of solvent B as follows: 10% B (3 min), 20% B (10 min), 30% B (30 min), 90% B (12 min), and 10% B (7 min). An injection volume of 20 μL was used, and the UV spectrum was monitored in the range of 190 nm to 600 nm. In addition to UV detection, mass spectrometry (MS) analysis was performed with an analysis time of 62 min. The scanning range for mass data acquisition was set to 90–1350 *m*/*z* in both positive and negative ionization modes. It should be noted that compound identifications were based on MS fragmentation patterns and database matching. Future studies should include confirmation using authentic standards, particularly for phenolic glycosides, where structural isomers are common and may affect computational predictions [29,60].

### 5.3. Database Preparation

The 3D structures of the identified compounds, as well as the known inhibitors for Hsp90: 17AAG, Geldanamycin and Radicicol for NTD binding site [61], Novobiocin [62] for CTD inhibition and Enniatin A [47] for the Middle-domain were obtained from PubChem database (PubChem ID list available in Supplementary Table S2) and converted to PDB format using Open Babel to ensure compatibility. The 3D structure of Hsp90α (PDB ID: 7KRJ) [63] was obtained from the RCSB Protein Data. As the structure lacked critical loops within the N-terminal domain required for molecular dynamics simulations, homology modeling was carried out using SWISS-MODEL [64] to reconstruct the missing regions. The modeling process was based on the amino acid sequence of Hsp90α (UniProt ID: P07900), with the 7KRJ structure used as a template. Structural quality was evaluated through a Ramachandran plot generated by the PROCHECK tool. The prepared protein structure was then processed using the Protein Preparation Wizard in Maestro (Schrödinger Suite), which included the removal of water molecules, addition of nonpolar hydrogens, creation of salt bridges, and assignment of appropriate charges. Ligands were prepared using the LigPrep module, which included the addition of missing hydrogens, bond order assignment, and energy minimization using the OPLS4 force field within the Schrödinger environment. All chemical structures were constructed and reoriented using ChemDraw Professional (version 23.1.1). All known chiral centers are represented with either dashes or wedges, while chiral centers with unknown absolute stereochemistry are represented with wavy bonds. Atomic labels used Arial 10 with 120° bond angles, 18% bond spacing, 0.2 in bond length, 0.0278 in bold width, and 0.0083 in line width. The figure was exported in 600 dpi resolution as a .cdx file.

### 5.4. Extra Precision Docking

Extra Precision (XP) docking mode in the Schrodinger Glide module was used to screen the identified compounds against the prepared structure of Hsp90α. The docking grids were generated using a targeted docking approach, where three receptor grid boxes were centered on the centroid of the binding pocket formed between the middle domains of the two chains of the protein dimer, the middle domain of chain A alone, and the C-terminal domain of the protein. The grid boxes were set to exclude the N-terminal domain, since we aimed to avoid screening ATP-competitive ligands and instead focus on compounds that may bind to alternative sites of Hsp90. Docking in XP mode was conducted with default parameters, including a van der Waals radius scaling factor set to 0.8 and a partial charge cutoff set to 0.15 for non-polar atoms. No constraints were applied during docking. The ligand poses were scored and ranked based on their Glide score. A comparative ranking was used to identify the top two ranking molecules per domain, which were selected for subsequent MD simulations. Ligand-protein interactions were visualized and analyzed using the Ligand Interaction module in Maestro to identify key residue interactions contributing to binding affinity.

### 5.5. Molecular Dynamics Simulation

The top two molecules showing the highest binding affinities with the middle domain interchain or chain A, and C-terminal domain of the protein were selected for molecular dynamics (MD) simulations. The docking-generated complexes were used as the initial conformation for the simulation system preparation using the System Builder wizard in Desmond.

The preparation consisted of embedding the Hsp90 dimer complex and ligand-bound complexes, in a TIP3P solvent using a Cubic box system with 10 Å padding, neutralizing the systems with Cl^−^ ions, then adding 0.15 M of NaCl concentration to mimic physiological ionic strength. The generated systems were then subjected to 100 ns MD simulations using OPLS4 force field, which is specifically optimized for proteins and drug-like ligands, including phytochemicals. The multigrator integrator was employed, using the Martyna-Tobias-Klein (MTK) method for pressure coupling and the Nose-Hoover thermostat for temperature regulation. The temperature was set to 300 K, and the pressure to 1.01325 bar, using relaxation times (τ) of 1.0 ps and 2.0 ps, respectively, for thermostat and barostat. A time step of 2 fs was used for bonded and short-range nonbonded interactions, and 6 fs for long-range interactions. Long-range electrostatics were handled using the u-series method, with a cutoff radius of 9.0 Å for non-bonded interactions. The constraints on bond lengths involving hydrogen atoms were applied using the default Desmond algorithm, with a convergence tolerance of 1 × 10^−8^ and a maximum of 16 iterations. Initial velocities were randomly assigned from a Maxwell–Boltzmann distribution at 300 K using a fixed seed (2007) to ensure reproducibility. Simulation trajectories were saved every 500 ps, and system energies were recorded every 1.2 ps. Checkpoint files were written every 240.06 ps, and simulation box dimensions were monitored at 1.2 ps intervals.

Following the completion of molecular dynamics (MD) simulations for each system, the resulting trajectories were analyzed using the Simulation Interaction Diagram (SID) in Maestro. This analysis quantified system dynamics through metrics such as root mean square deviation (RMSD), root mean square fluctuation (RMSF). The average (Avg) and standard deviation (SD) of each parameter were calculated. The protein-ligand interactions (PLI) were tracked throughout the simulation for each residue. Additionally, the Prime Molecular mechanics with generalized Born and surface area solvation (MM/GBSA) was calculated to evaluate the free binding affinity between the protein and selected molecules during the last 20 ns of the MD trajectory, to further evaluate the modulatory effect of said molecules on Hsp90α.

### 5.6. ADMET Assessment

The top hit compounds were assessed for ADME parameters, pharmacokinetic properties, druglike nature, and medicinal chemistry friendliness using SwissADME and ProTox 3.0.

## Figures and Tables

**Table 1:** Identified compounds in each extract of *M. vulgare* along with their molecular weight.

**Organic extract**	**Methanol**	**Identified Compound**	**Molecular Weight**
		Heneicosane	296.57
		Phytol	296.54
		Heptadecane	240.47
		Luteolin 7-o-glucuronide	462.36
		Rosmarinic acid	360.31
		Marrubiin	332.44
		3-Deoxo-15(S)-methoxyvelutine	536.7
		Syringic acid	198.2
		Apigenin 7-[2-glucuronosyllactate]	494.4
		Apigenin 7-O-glucuronide	494.4
	**Dichloromethane**	**Identified compound**	**Molecular weight**
		Octadeca-2,4,6-trienoic acid	278.43
		Marrubiin	332.44
		Luteolin	286.25
		Rosmarinic acid	360.31
		Acacetin	284.3
		Diosmetin	300.3
		Ladanein	300.3
		Luteolin	286.2
		Apigenin 7-acetate	328.3
		Naringenin	272.3
	**Ethanol**	**Identified compound**	**Molecular weight**
		11-oxomarrubiin	520.7
		3-hydroxyapigenin 4′-O-(6′′-O-p-coumaroyl)-glucoside	610.6
		Acteoside	624.6
		Alyssonoside	610.6
		Apigenin 4′-O-(6′′-O-p-coumaroyl)-glucoside	610.6
		Apigenin 6,8-di-C-glucoside (Vicenin II)	594.5
		Apigenin 7-[2-glucuronosyllactate]	494.4
		Apigenin 7-O-(4′′-p-coumaroyl)-glucoside (Terniflorin)	610.6
		Apigenin 7-O-(6′′-p-coumaroyl)-glucoside	610.6
		Apigenin 7-O-glucuronide	494.4
		Arenarioside	610.6
		Ballotetroside	610.6
		Chlorogenic acid	354.3
		Cyllenin A	520.7
		Diosmetin	300.3
		Echinacin	578.52
		Forsythoside B	624.6
		Heptadecane	240.47
		Ladanein	300.3
		Leucosceptoside A	610.6
		Luteolin	286.2
		Luteolin 7-acetate	344.3
		Luteolin 7-o-glucuronide	462.36
		Luteolin 7-O-rutinoside	594.5
		Marrubenol	336.47
		Marrubiin	332.44
		Marruboside	610.6
		Marruliba-acetal	520.7
		Oxacyclohexadecan-2-one	240.38
		Polyodonine	520.7
		Preleosibirin	520.7
		Premarrubiin	332.43
		Quercetin 3-O-rutinoside (Rutin)	610.5
		Rosmarinic acid	360.3
		Samioside	610.6
		Syringic acid	198.2
		Verbascoside	624.59
		Vicenin 2	594.52
	**Ethyl Acetate**	**Identified compound**	**Molecular weight**
		Acacetin	284.3
		Apigenin 7-acetate	328.3
		Diosmetin	300.3
		Diosmetin-7-O-glucoside	434.4
		Docosane	310.60
		Galangin	270.2
		Ladanein	300.3
		Marrubenol	332.5
		Marrubic acid	332.5
		Marrubiin	332.44
		Methyl linoleate	294.47
		Peregrinin	332.5
		Peregrinol	332.5
		Premarrubiin	332.43
		Rosmarinic acid	360.31
		Stearyl alcohol	270.49
		Vulgarol	332.5
	**Hexane**	**Identified compound**	**Molecular weight**
		Chlorogenic acid	354.3
		Caffeoylmalic acid	266.2
		Aesculin	340.3
		Ladanein	314.29
		Methyl linoleate	294.47
		Octadeca-2,4,6-trienoic acid	278.43
		Premarrubiin	332.43
		Rosmarinic acid	360.31
		Cis-Piperitone oxide	166.1
**Aqueous extract**		**Identified compound**	**Molecular weight**
		Eugenol	164.20
		Para-coumaric acid	164.16
		Stigmast-5-en-3-ol	414.71
		Β-sitosterol	414.71
		Oxacyclohexadecan-2-one	240.38
		Hexadecane	226.45
		O-Coumaric acid	164.2
		P-Coumaric acid	164.2
		Apigenin 7-acetate	328.3

**Table 2:** Docking scores of the identified compounds from M. vulgare against the middle-domain chain A, interchain region, and C-terminal domain (CTD) of Hsp90α. Ligand poses were scored and ranked based on Glide score from the Glide XP docking mode.

Middle Domain Chain A		Middle Domain Interchain		CTD	
**Compound**	**Docking Score**	**Compound**	**Docking Score**	**Compound**	**Docking Score**
Forsythoside B	−6.84	Forsythoside B	−7.96	Luteolin	−7.82
Terniflorin	−6.59	Samioside	−7.64	Naringenin	−7.50
Rosmarinic_acid	−6.48	Terniflorin	−7.44	Chlorogenic acid	−7.33
Luteolin7_o_glucuronide	−6.48	Alyssonoside	−7.33	Galangin	−7.20
Apigenin	−6.40	Verbascoside	−6.93	Forsythoside B	−6.99
Samioside	−6.26	Acteoside	−6.93	Diosmetin	−6.91
Acteoside	−5.97	Rosmarinic_acid	−6.85	Terniflorin	−6.89
Verbascoside	−5.97	LeucosceptosideA	−6.71	Samioside	−6.80
Luteolin7_O_rutinoside	−5.91	Echinacin	−6.50	Acacetin	−6.65
Vicenin2	−5.90	Vicenin2	−6.20	Vulgarin	−6.52
Echinacin	−5.86	Diosmetin	−6.06	Ladanein	−6.43
Rutin	−5.73	Syringic_acid	−6.03	Rosmarinic_acid	−6.33
Alyssonoside	−5.69	Luteolin	−5.94	Alyssonoside	−6.26
Luteolin	−5.62	Naringenin	−5.77	Acteoside	−6.26
Aesculin	−5.56	Diosmetin_7_O_glucoside	−5.70	Verbascoside	−6.26
Eugenol	−5.38	Acacetin	−5.57	cis_Piperitone	−6.13
Diosmetin	−5.18	Luteolin7_O_rutinoside	−5.53	Echinacin	−5.71
Chlorogenic acid	−4.95	Rutin	−5.49	Syringic_acid	−5.64
Acacetin	−4.90	Ladanein	−5.41	Aesculin	−5.63
Galangin	−4.88	Galangin	−5.34	Luteolin7_O_rutinoside	−5.60
Vulgarin	−4.86	Chlorogenic acid	−5.05	Apigenin	−5.59
Syringic_acid	−4.71	Aesculin	−4.99	Rutin	−5.42
Ladanein	−4.62	Novobiocin	−4.98	LeucosceptosideA	−5.36
Peregrinin	−4.61	Apigenin	−4.92	Diosmetin_7_O_glucoside	−5.27
Diosmetin_7_O_glucoside	−4.54	Eugenol	−4.87	Eugenol	−5.24
Marrubiin	−4.48	Vulgarin	−4.85	Marrubenol	−4.91
o_Coumaric_acid	−4.48	Peregrinin	−4.82	Vicenin2	−4.78
Oxacyclohexadecan_2_one	−4.44	o_Coumaric_acid	−4.77	Luteolin7_o_glucuronide	−4.63
Para_coumaric_acid	−4.44	Oxacyclohexadecan_2_one	−4.57	Para_coumaric_acid	−4.46
Cyllenin_A	−4.35	Luteolin7_o_glucuronide	−4.35	Radicicol	−4.43
Naringenin	−4.26	Cyllenin_A	−4.26	o_Coumaric_acid	−4.16
cis_Piperitone	−4.25	Para_coumaric_acid	−4.19	17AAG	−4.14
Caffeoylmalic acid	−4.08	Marrubiin	−4.13	Oxacyclohexadecan_2_one	−3.88
Novobiocin	−3.99	Marrubenol	−4.10	Novobiocin	−3.69
Radicicol	−3.86	cis_Piperitone	−4.10	Premarrubiin	−3.56
Premarrubiin	−3.79	Enniatin A	−4.05	Caffeoylmalic acid	−3.52
17AAG	−3.47	Radicicol	−3.99	Marrubiin	−3.50
Marrubenol	−3.30	Premarrubiin	−3.92	Cyllenin_A	−3.42
Geldanamycin	−3.02	24_Ethylcholest_5_en_3beta_ol	−3.81	Peregrinin	−3.39
Leucosceptoside A	−2.74	Sitosterol	−3.81	24_Ethylcholest_5_en_3beta_ol	−3.13
Enniatin A	−2.50	Stigmast_5_en_3_ol	−3.81	Sitosterol	−3.13
24_Ethylcholest_5_en_3beta_ol	−2.23	Caffeoylmalic acid	−3.61	Stigmast_5_en_3_ol	−3.13
Sitosterol	−2.23	Geldanamycin	−3.43	Geldanamycin	−2.96
Stigmast_5_en_3_ol	−2.23	Phytol	−1.39	Phytol	−2.37
Phytol	−0.58	Methyl_linoleate	0.14	Enniatin A	−2.29
		Octadecatrienic_acid	0.86	Methyl_linoleate	−1.00
				Octadecatrienic_acid	−0.56

**Table 3:** The averages (avg) and standard deviations (SD) of the parameters generated from molecular dynamics of the simulated systems.

		Unbound Structure	Middle Domain Chain A		Middle Domain Interchain	CTD	
**Compounds**		Hsp90α alone	Forsythoside B	Rosmarinic Acid	Forsythoside B	Chlorogenic acid	Luteolin
**RMSD (Å)**	Avg	6.56	5.25	6.14	6.47	6.16	6.11
	SD	1.01	0.68	0.96	1.14	0.97	0.85
**RMSF (Å)**	Avg	2.65	2.18	2.40	2.25	2.52	2.54
	SD	2.86	2.23	2.66	2.31	2.70	2.79

**Table 4:** MM/GBSA analysis of the interaction between Forsythoside B bound to the middle domain interchain and Chlorogenic acid bound to the CTD of Hsp90α.

Complex	ΔG Average (kcal mol^−1^)	ΔG Standard Deviation	ΔG Range
**Forsythoside B (Interchain)**	−71.68	14.62	−107.08 to 0.69
**Chlorogenic Acid (CTD)**	−51.50	10.02	−64.47 to 0.53

**Table 5:** ADMET analysis of the hit compounds Chlorogenic acid and Forsythoside B. Data predictions were performed using SwissADME.

ADMET Properties			
**Chlorogenic acid**	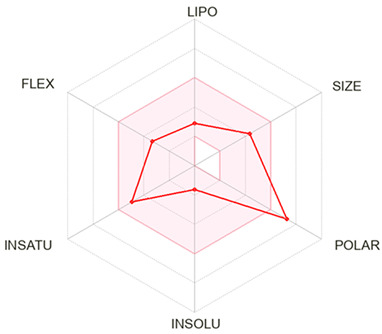	**Formula**	C16H18O9
		**Molecular weight**	354.31 g/mol
		**Druglikeness** **(Lipinski)**	Yes; 1 violation: NHorOH > 5
		**Log S (ESOL)**	−1.62
		**Consensus LogP_o/w_**	−0.38
		**TPSA**	164.75 Å^2^
		**Bioavailability Score**	0.11
**Forsythoside B**	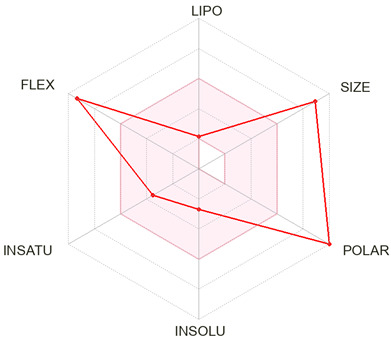	**Formula**	C34H44O19
		**Molecular weight**	756.70 g/mol
		**Druglikeness** **(Lipinski)**	No; 3 violations: MW > 500, NorO > 10, NHorOH > 5
		**Log S (ESOL)**	−2.69
		**Consensus LogP_o/w_**	−1.49
		**TPSA**	304.21 Å^2^
		**Bioavailability Score**	0.17

## Data Availability

The data supporting the findings of this study are included in the Supplementary Materials. Additional details are available from the corresponding author upon request.

## Supplementary Materials

The following supporting information can be downloaded at: https://www.mdpi.com/article/10.3390/ijms262412150/s1.

## Abbreviations

The following abbreviations are used in this manuscript:

CTD	C Terminal Domain
HPLC-MS	High-Performance Liquid Chromatography–Mass Spectormetry
Hsp90	Heat-Shock Protein 90
*M. vulgare*	*Marrubium vulgare*
MD	Molecular Dynamics
MMGBSA	Molecular mechanics with generalized Born and surface area solvation
NTD	N Terminal Domain
RMSD	Root Mean Square Deviation
RMSF	Root Mean Square Fluctuation
XP	Extra Precision
