# Supplementary material for: Phytochemical Profiling and Structure-Based Computational Characterization of Marrubium vulgare L. Compounds as Hsp90 Modulators
Source: Int J Mol Sci. 2025 Dec 17;26(24):12150. doi: 10.3390/ijms262412150 (PMC12733462; doi:10.3390/ijms262412150)
Supplement: Supplementary file 1 [file ijms-26-12150-s001.zip › Table S1_MS spectrums.pdf]

Organic solvent: Methanol		
**Compound**	**Molecular weight**	**Ms spectrum**
**Heneicosane**	296.57	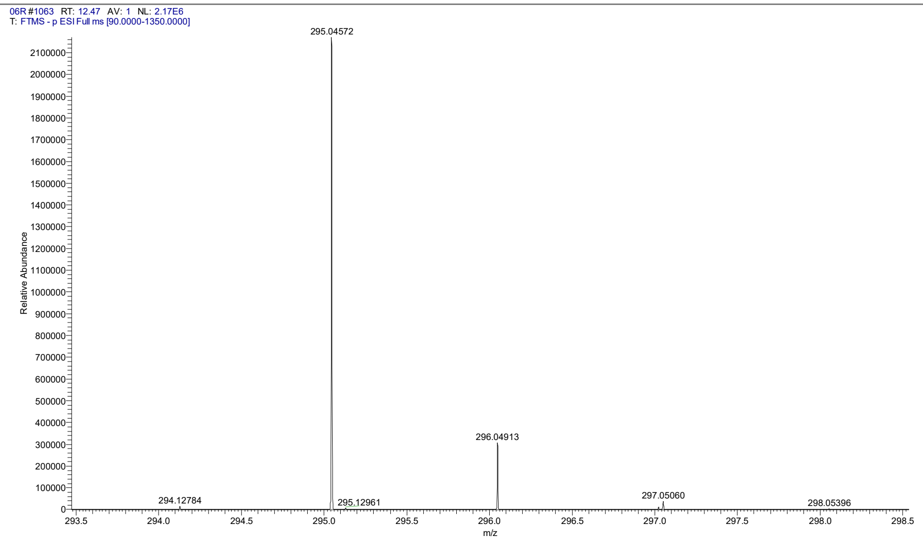
**Phytol**	296.54	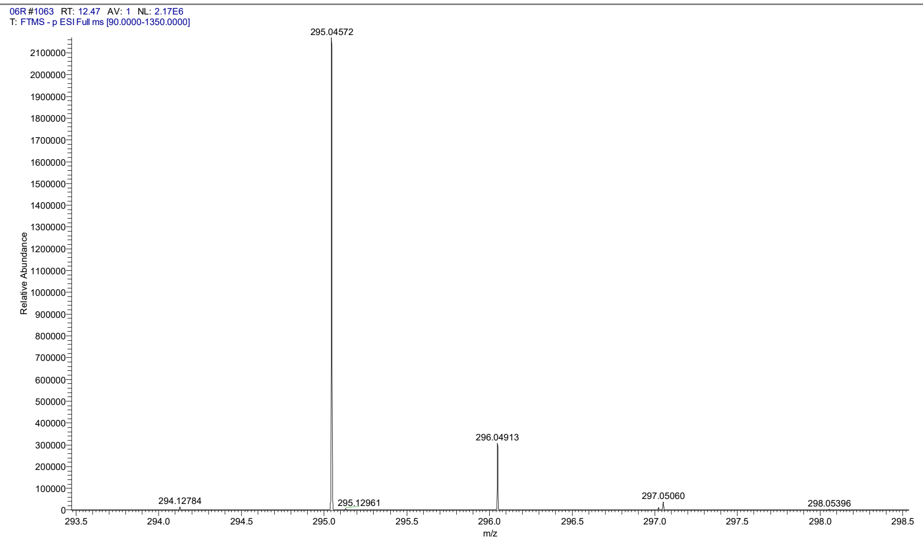

Heptadecane 240.47

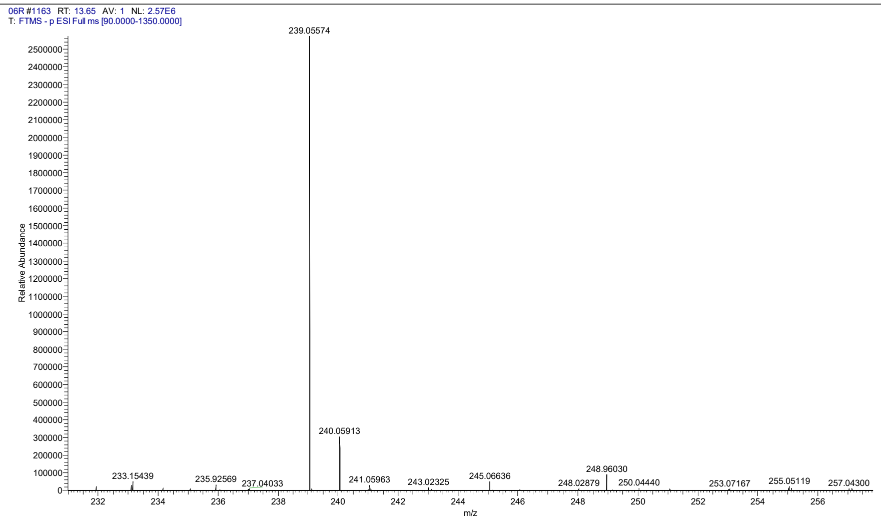

Luteolin 7-o-glucuronide 462.36

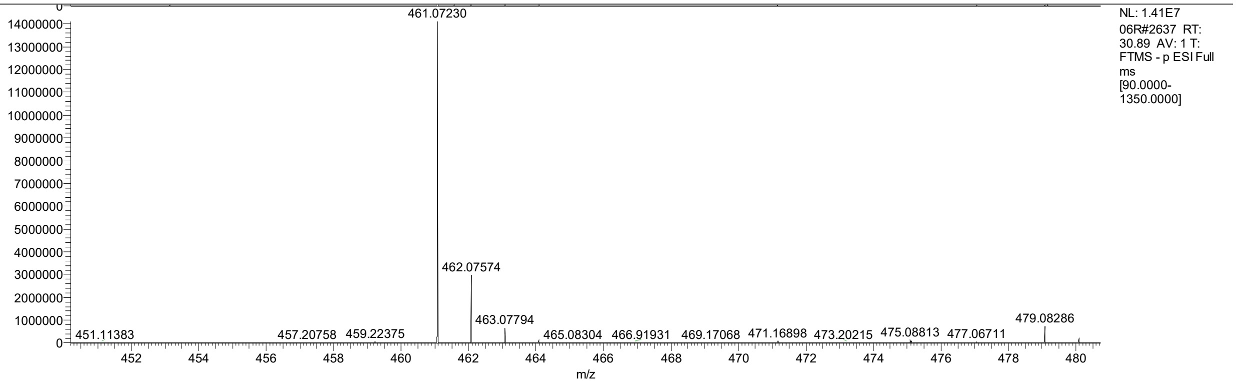

Rosmarinic acid 360.31

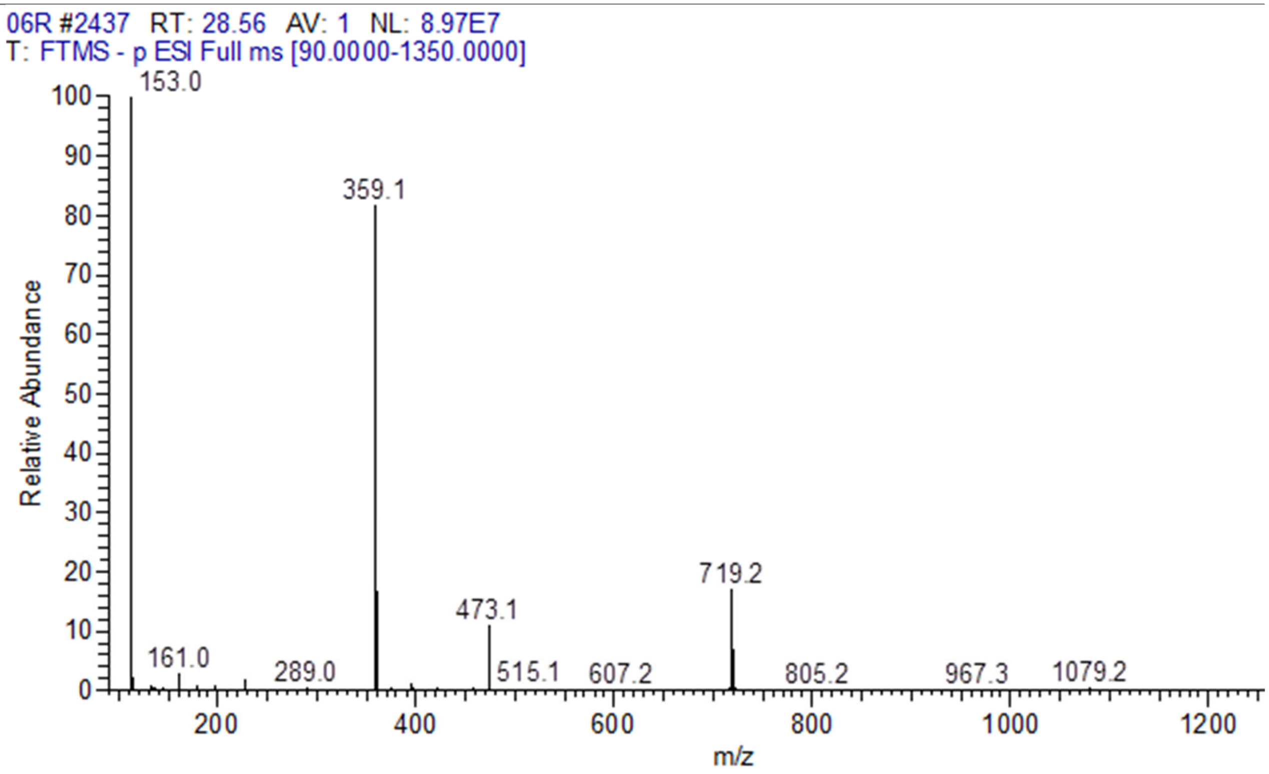

Marrubiin 536.7

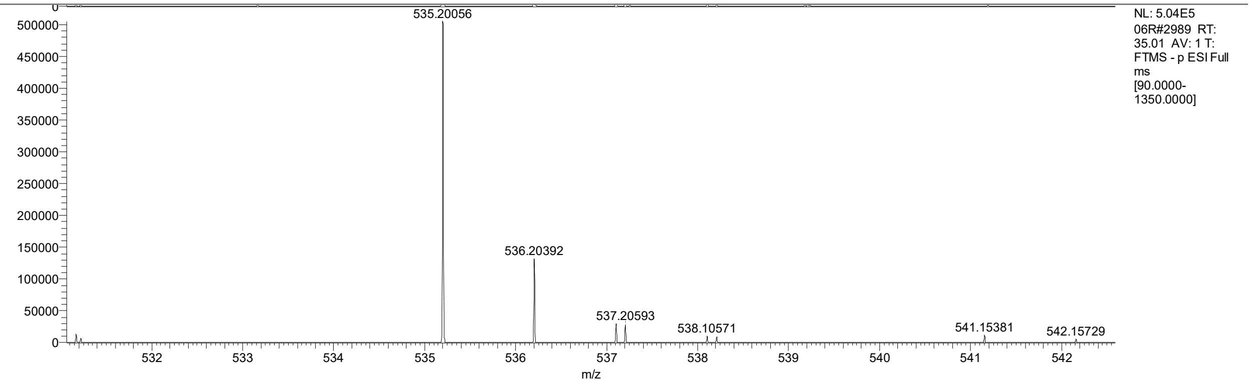

**3-Deoxo-15(S)-methoxyvelutine**	536.7	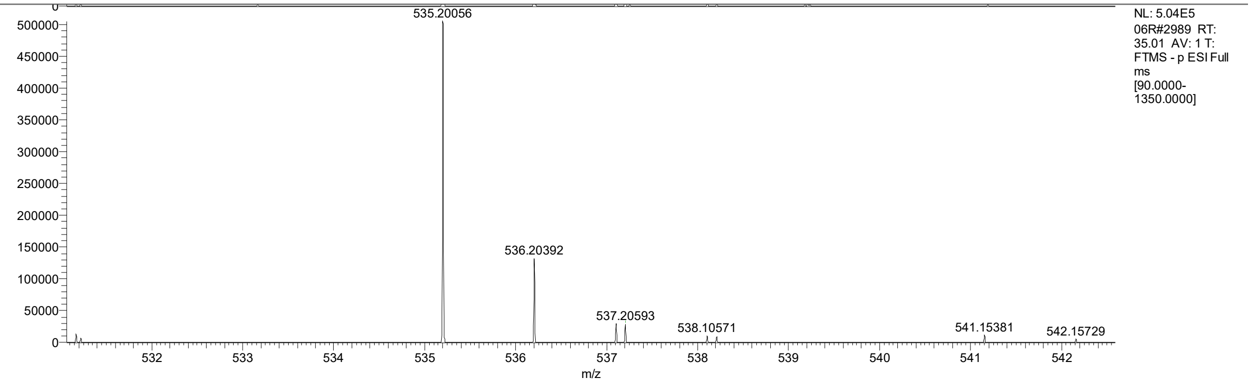
**Syringic acid**	198.2	
**Apigenin 7-[2-glucuronosyllactate]**	494.4	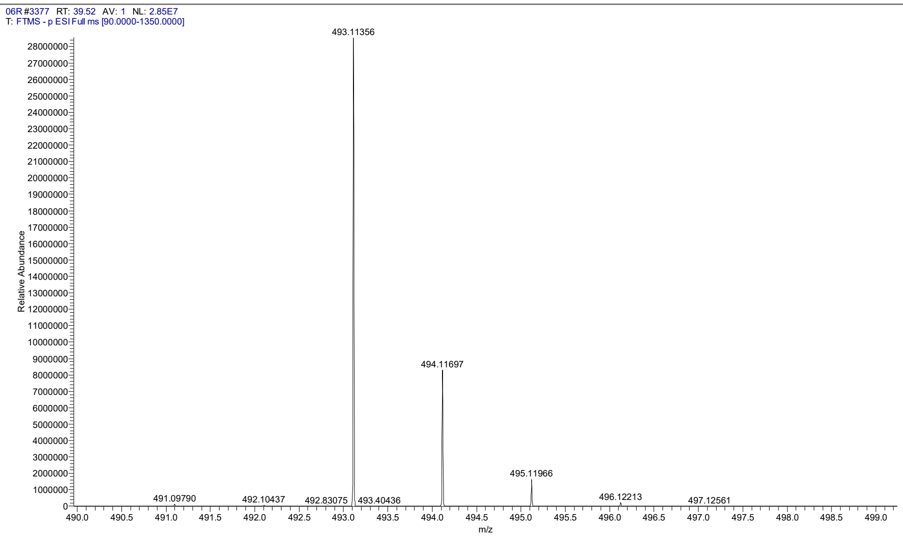

Apigenin 7-O-glucuronide

494.4

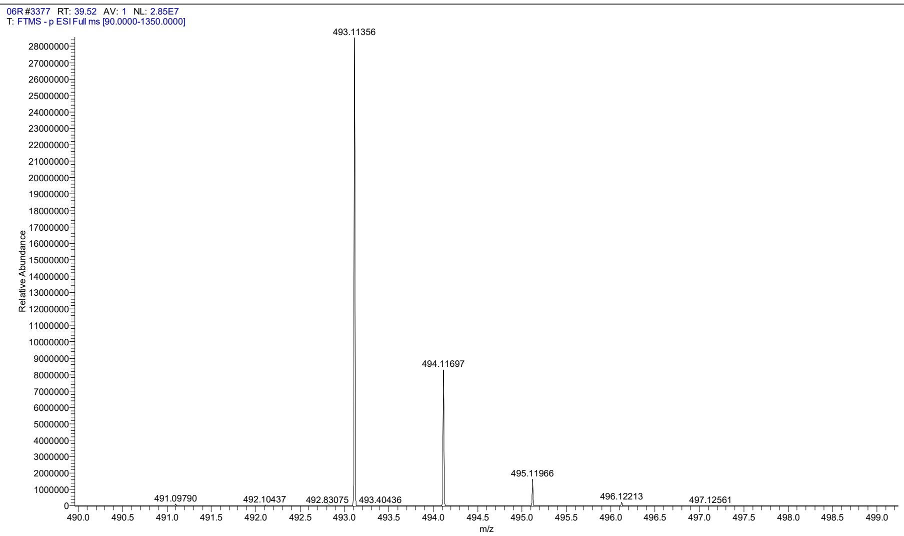

Organic solvent: Dichloromethane		
**Compound**	**Molecular weight**	**Ms spectrum**
**Octadeca-2,4,6-trienoic acid**	278.43	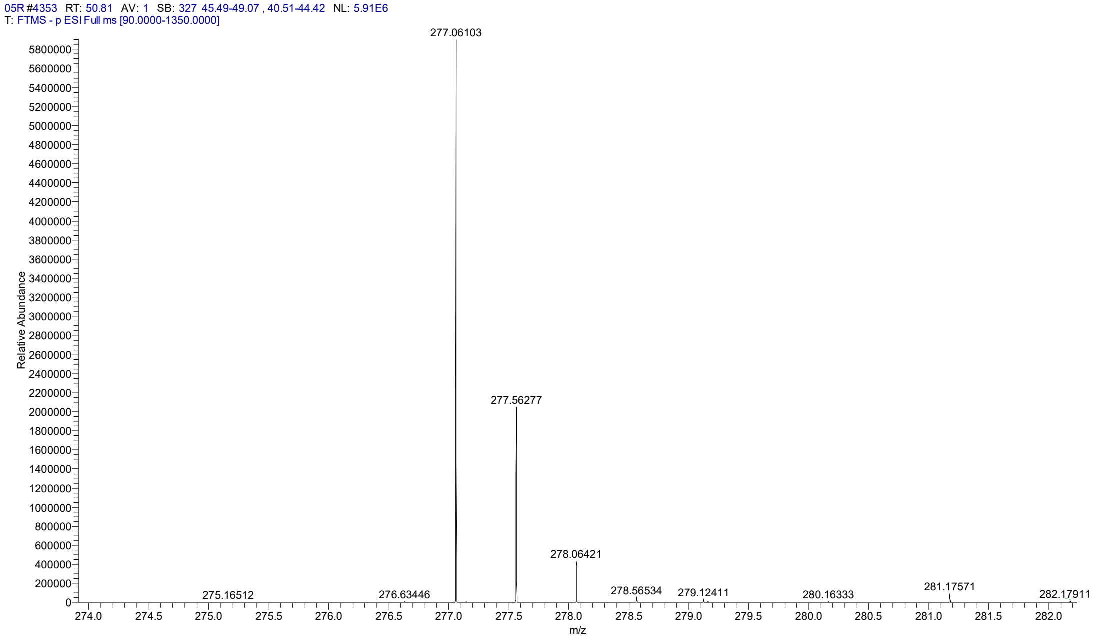

Marrubiin 332.44

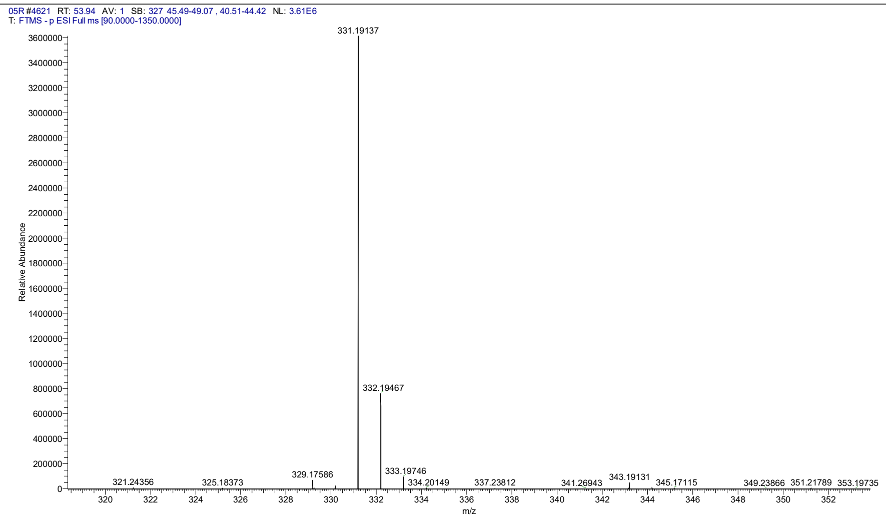

LUTEOLIN 286.25

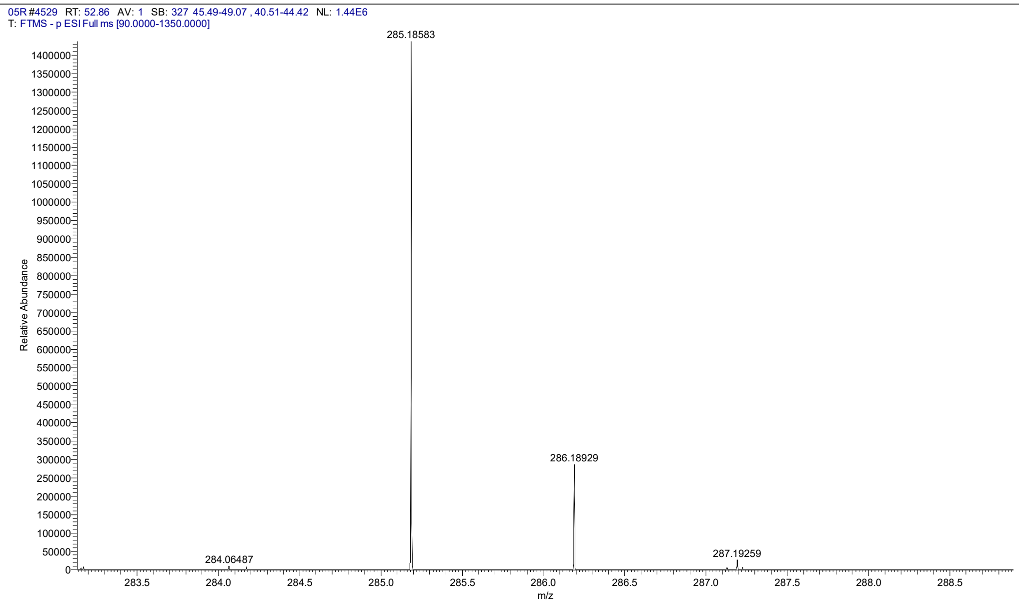

**Rosmarinic acid**	360.31	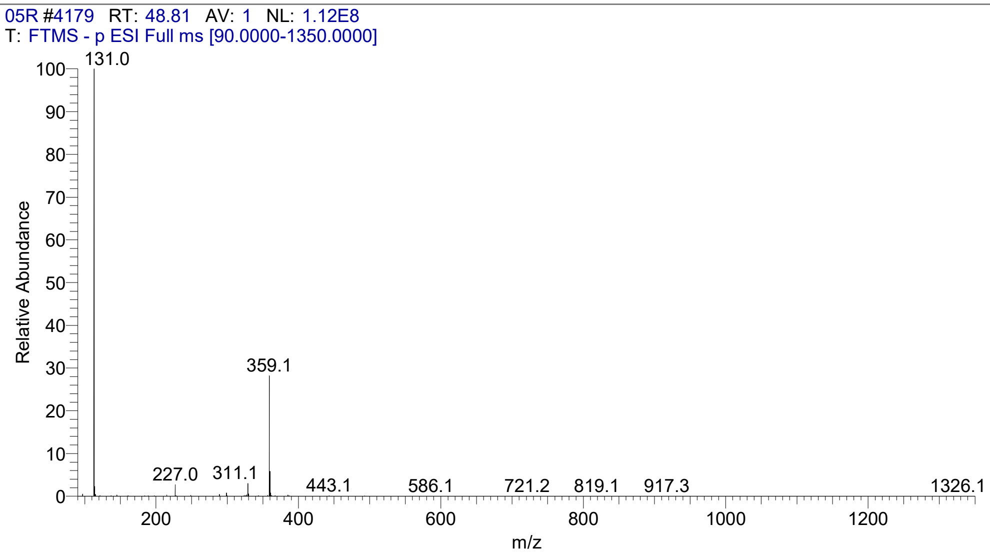
**Acacetin**	284.3	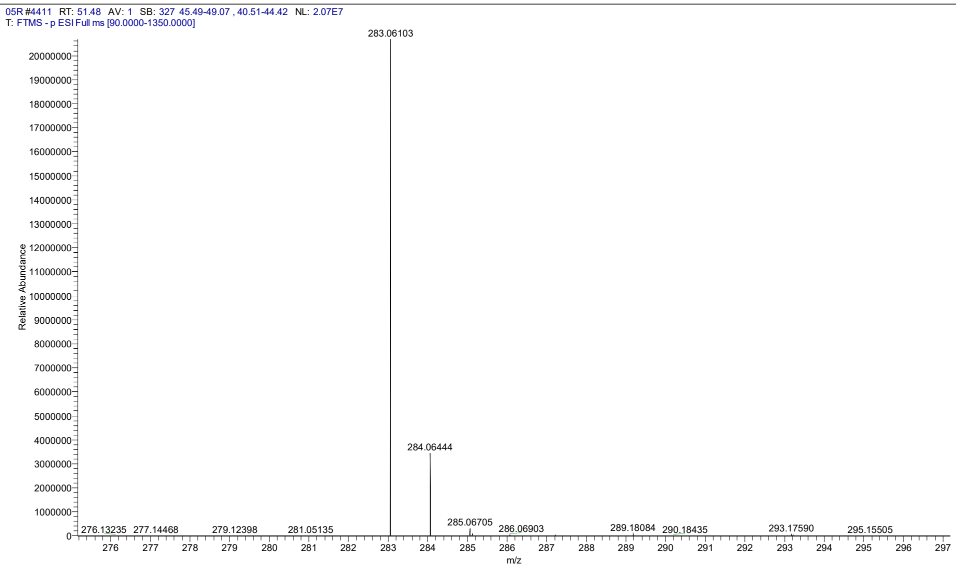

**Diosmetin**	300.3	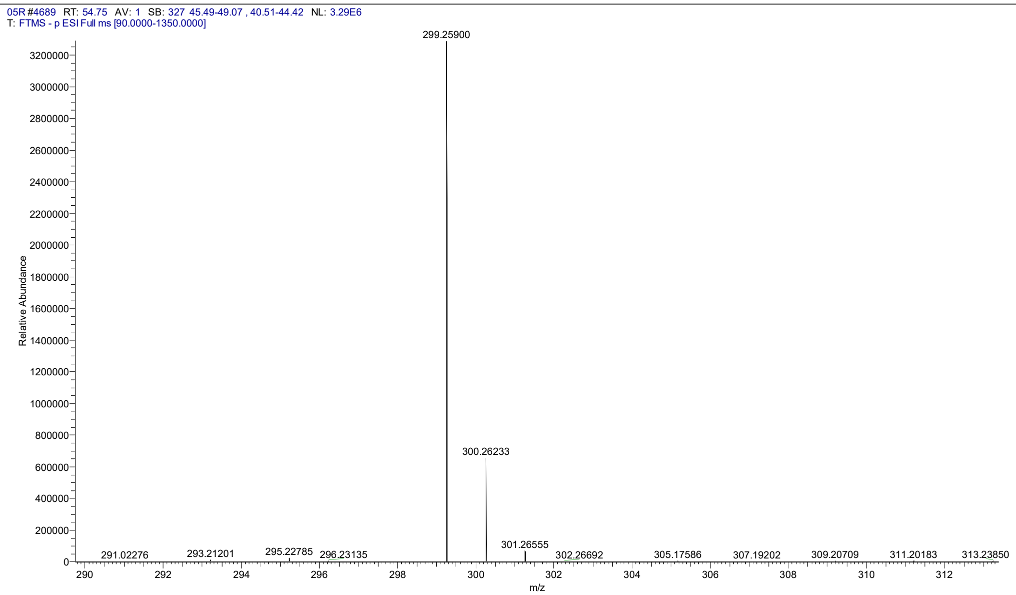
**Ladanein**	300.3	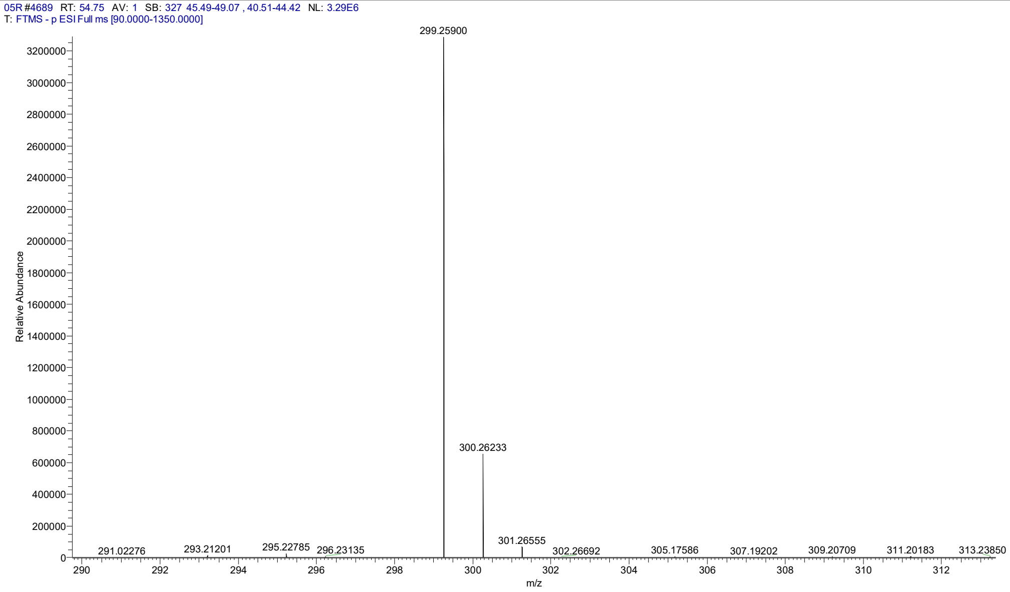

**Luteolin**	286.2	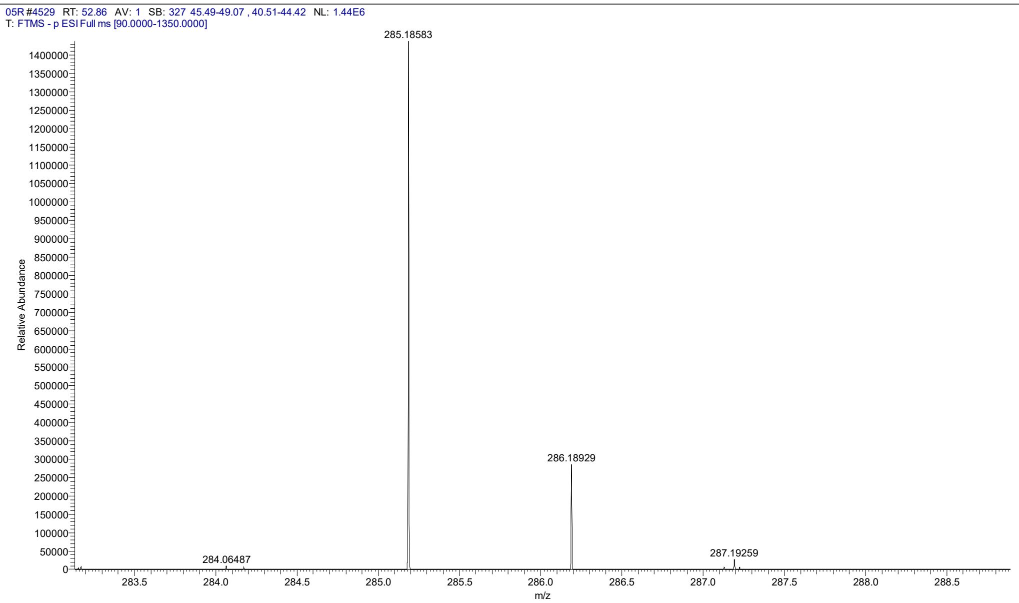
**Apigenin 7-acetate**	328.3	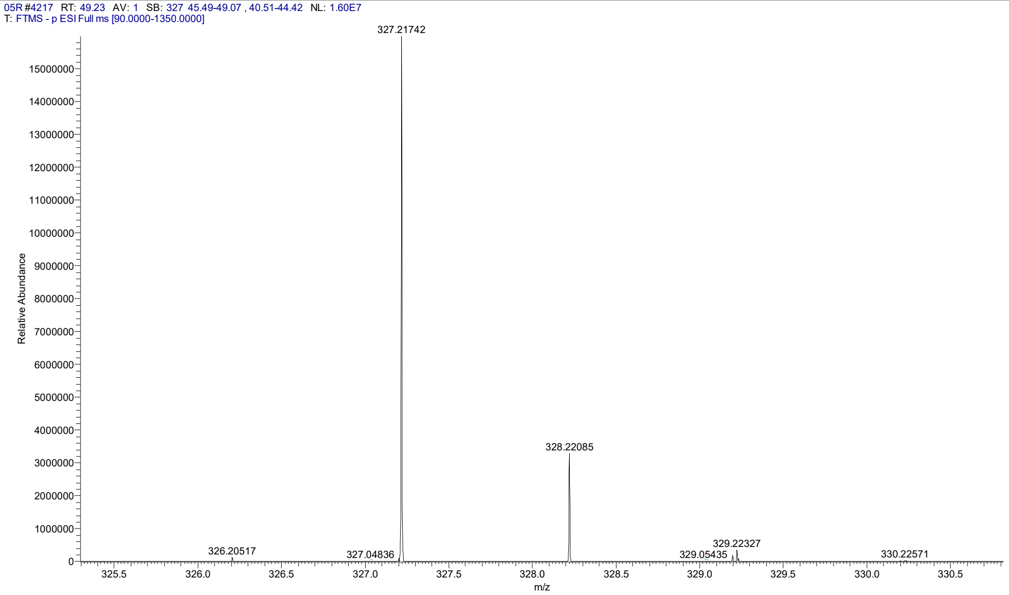

Naringenin 272.3

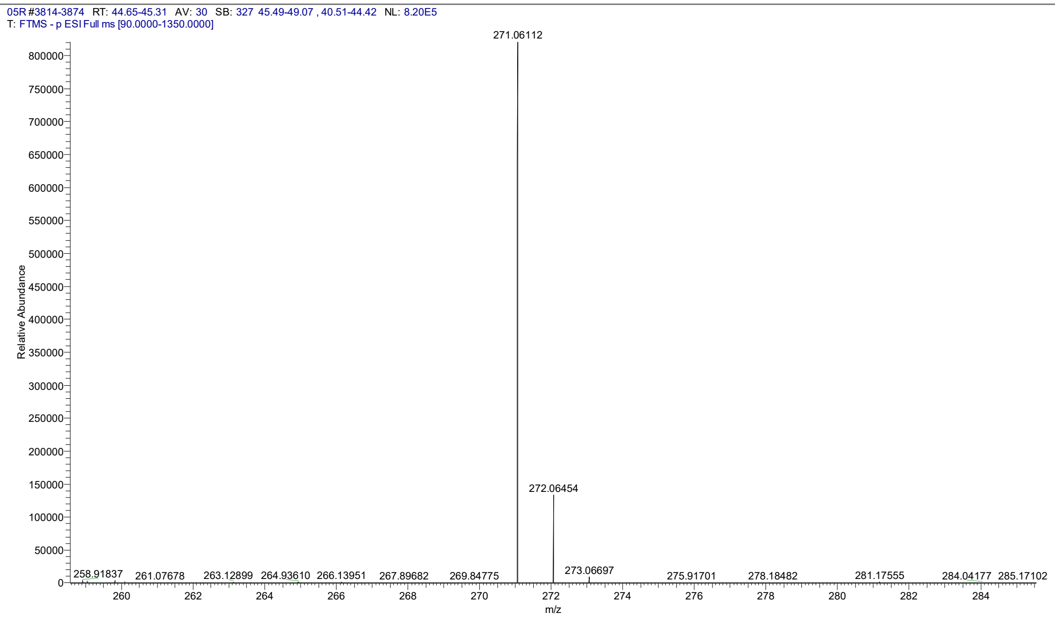

Organic solvent: Ethanol

Compound	Molecular weight	Ms spectrum
**11-Oxomarrubiin**	520.7	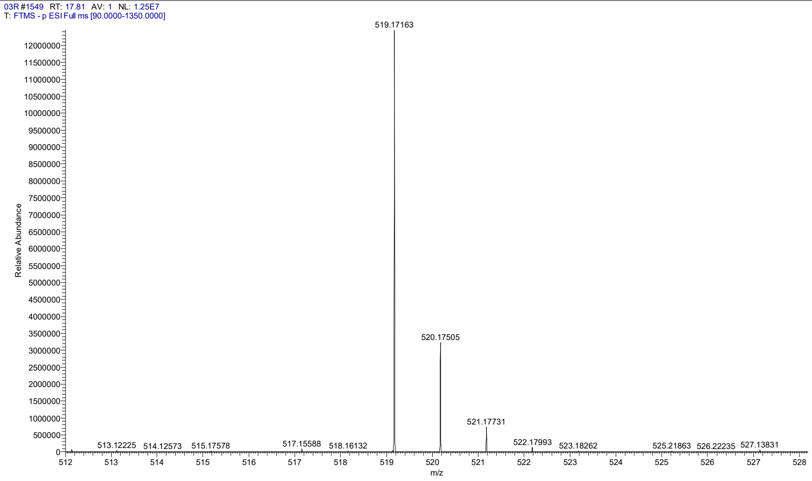
**3-hydroxyapigenin 4'-O-(6''-O-p-coumaroyl)-glucoside**	610.6	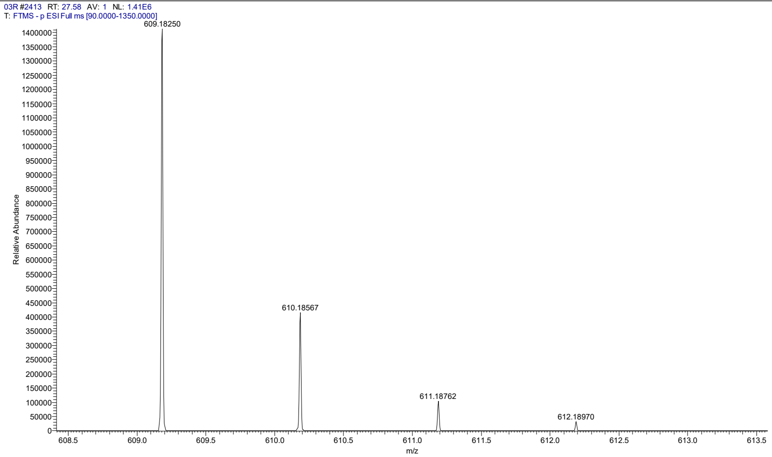

Acteoside 624.6

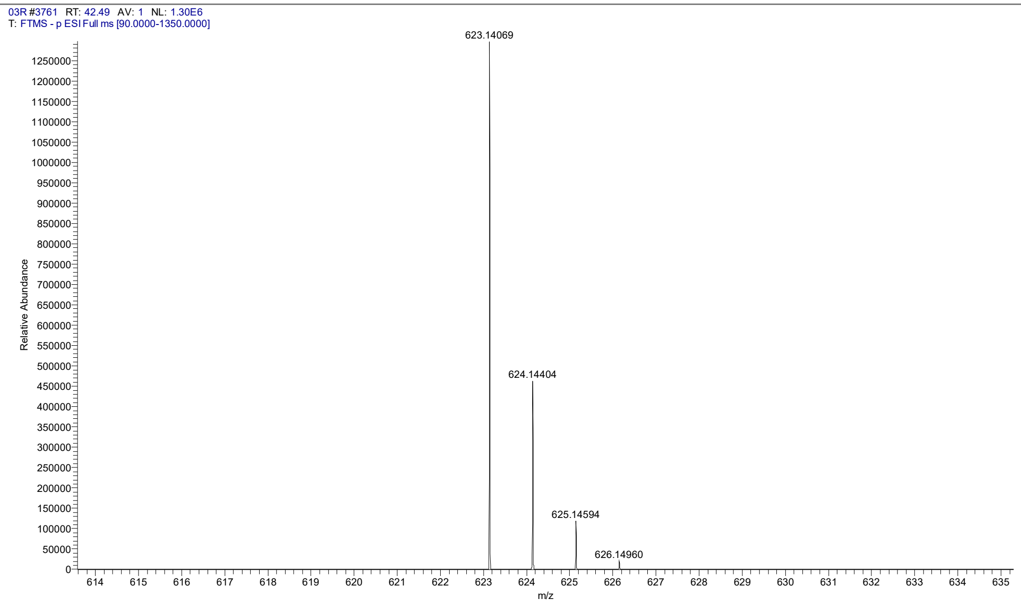

Alyssonoside 610.6

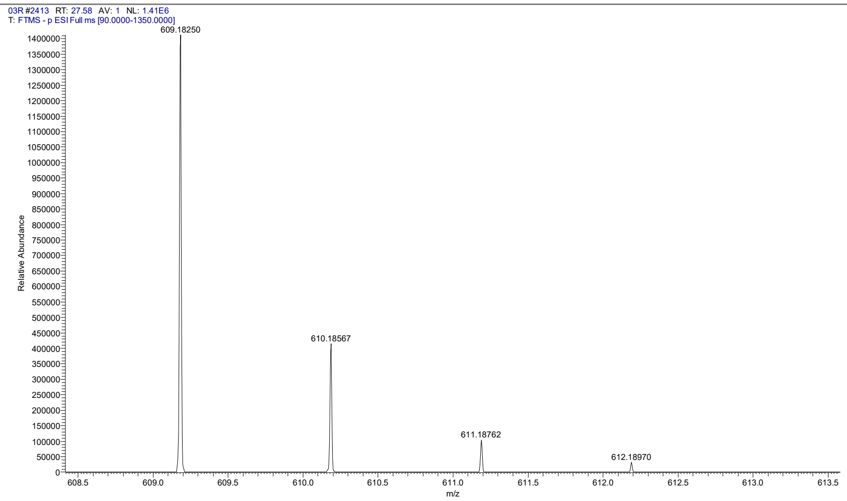

Apigenin 4'-O-(6''-O-p-coumaroyl)-glucoside

610.6

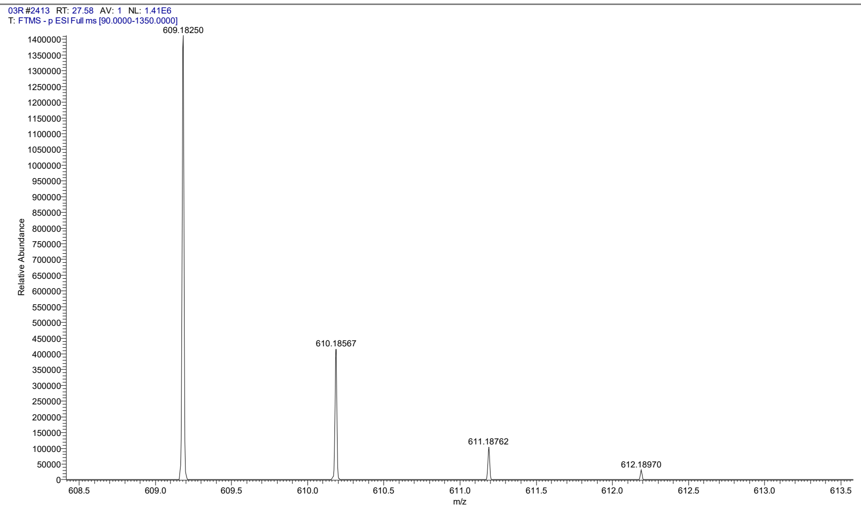

Apigenin 6,8-di-C-glucoside (Vicenin II)

594.5

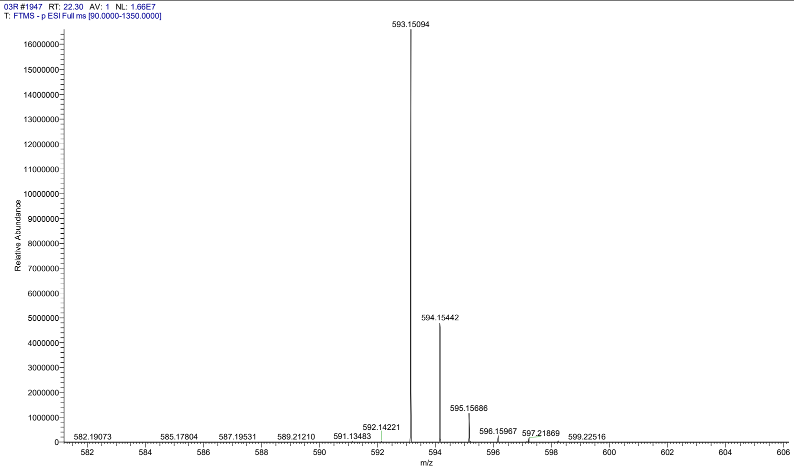

**Apigenin 7-[2-glucuronosyllactate]**	494.4	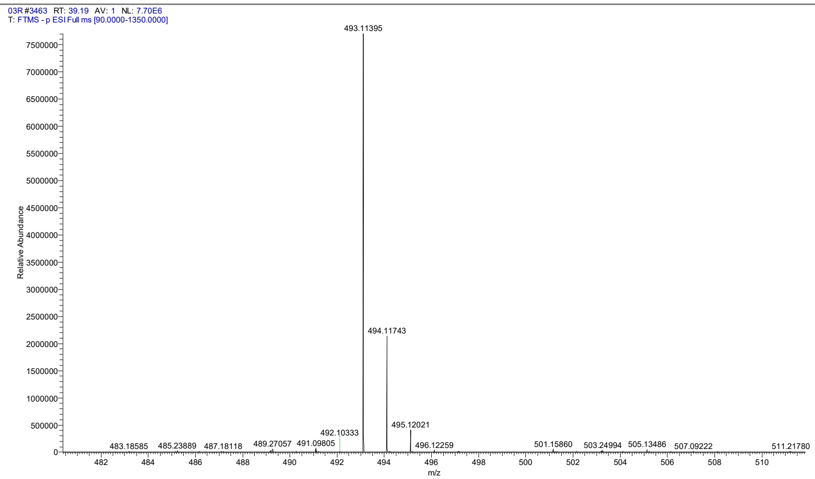
**Apigenin 7-O-(4''-p-coumaroyl)-glucoside (Terniflorin)**	610.6	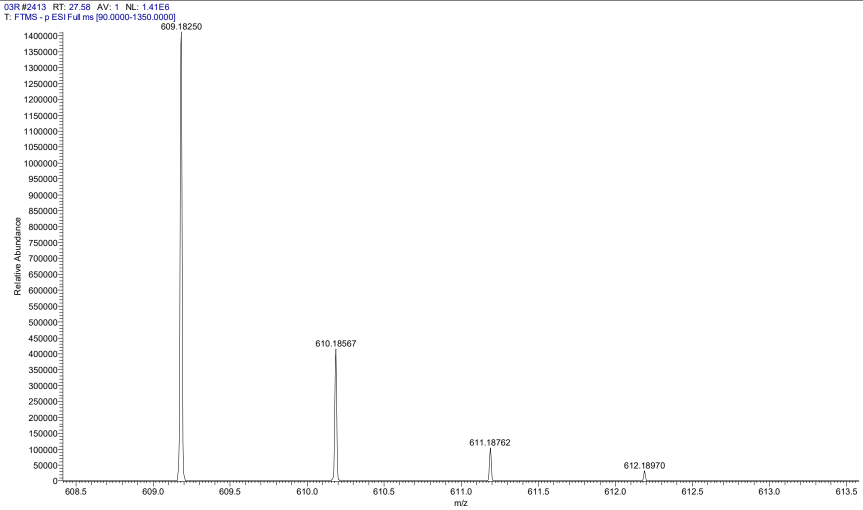

**Apigenin 7-O-(6''-p-coumaroyl)-glucoside**	610.6	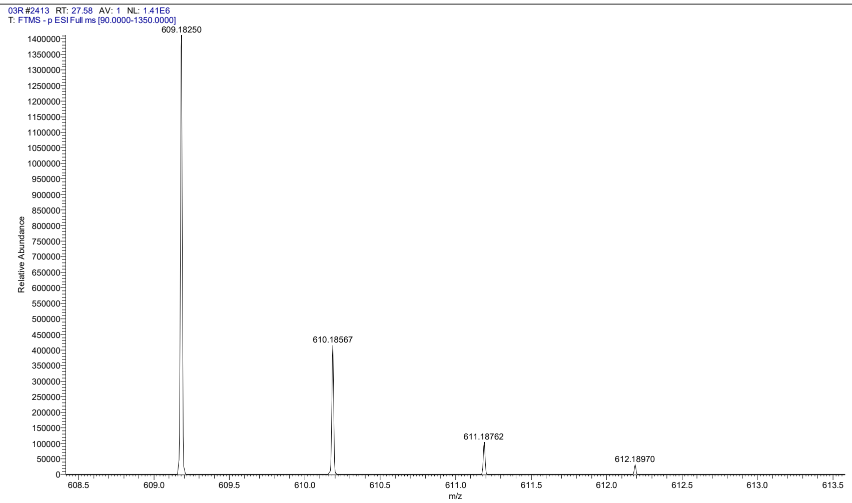
**Apigenin 7-O-glucuronide**	494.4	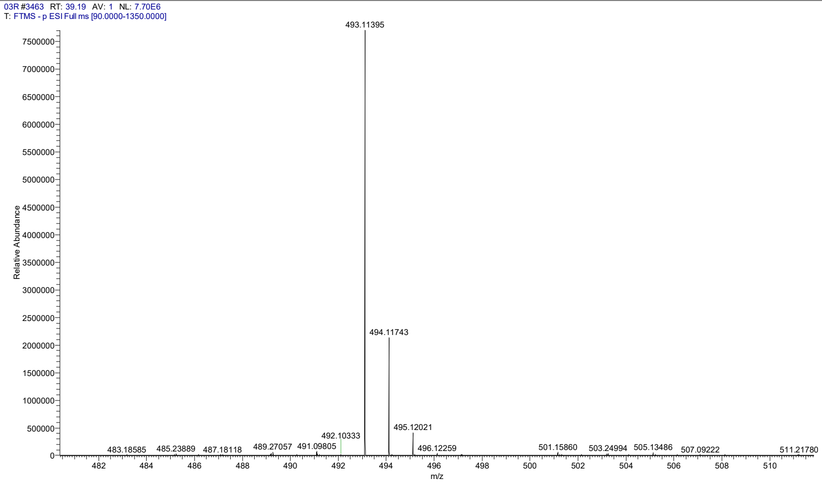

**Arenarioside**	610.6	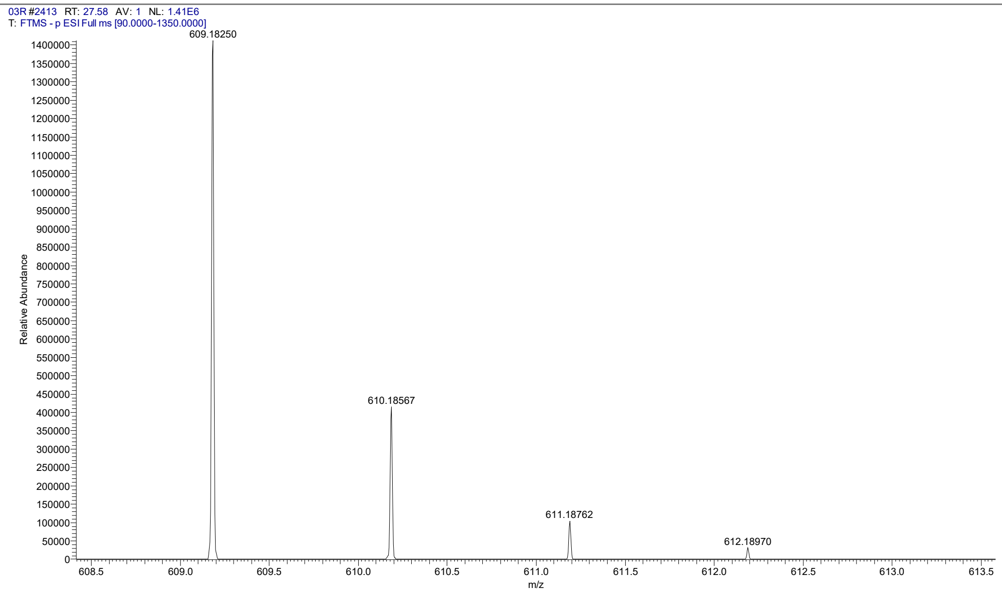
**Ballotetroside**	610.6	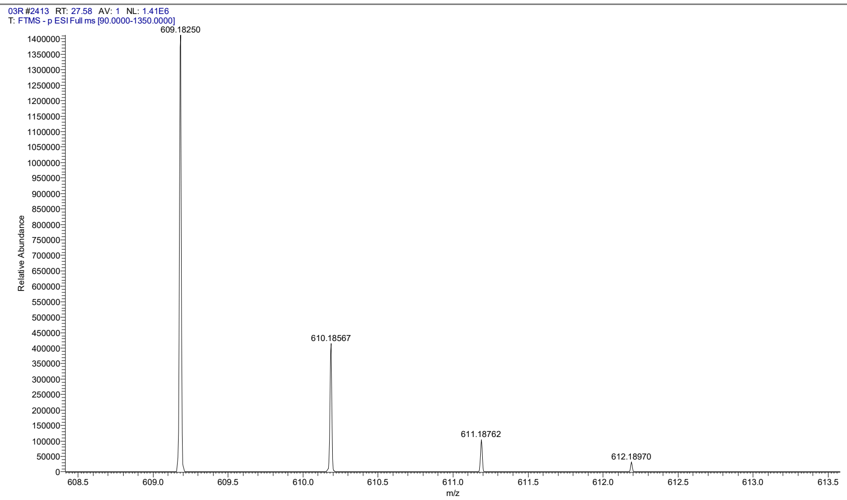

**Chlorogenic acid**	354.3	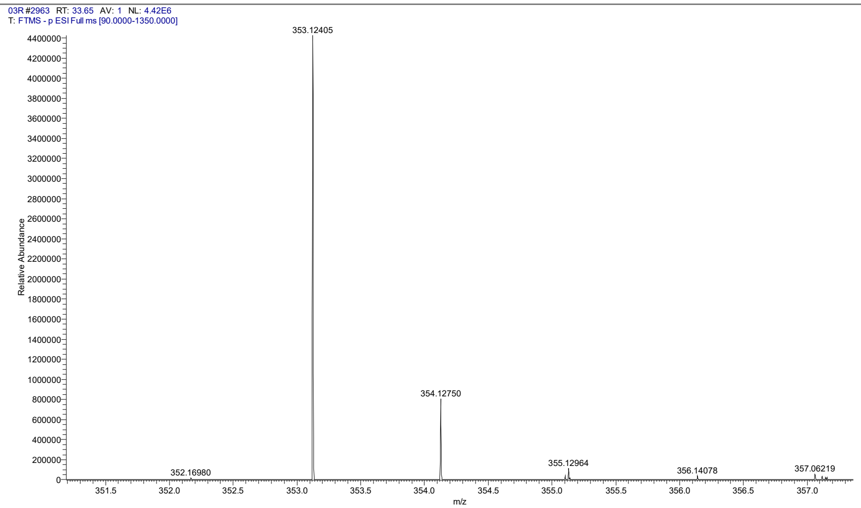
**Cyllenin A**	520.7	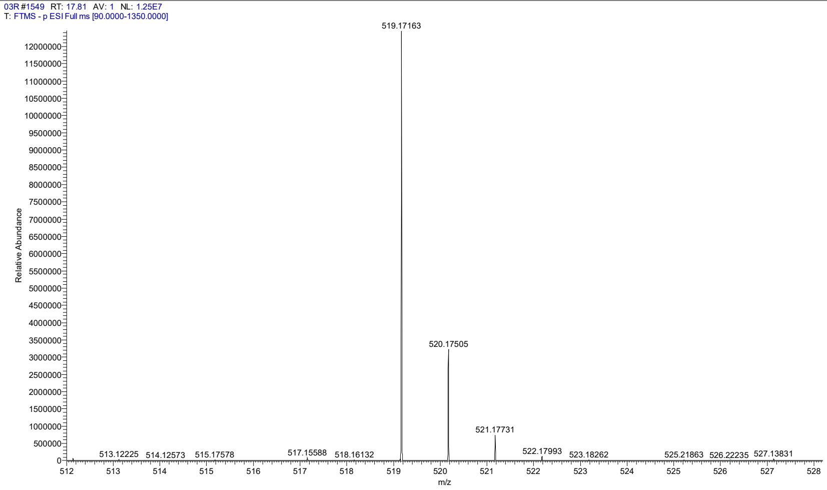

Diosmetin 300.3

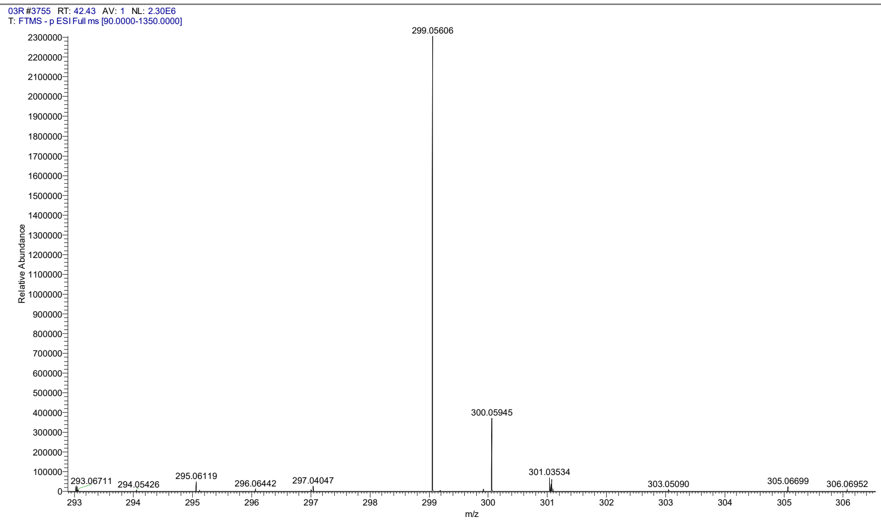

Echinacin 578.52

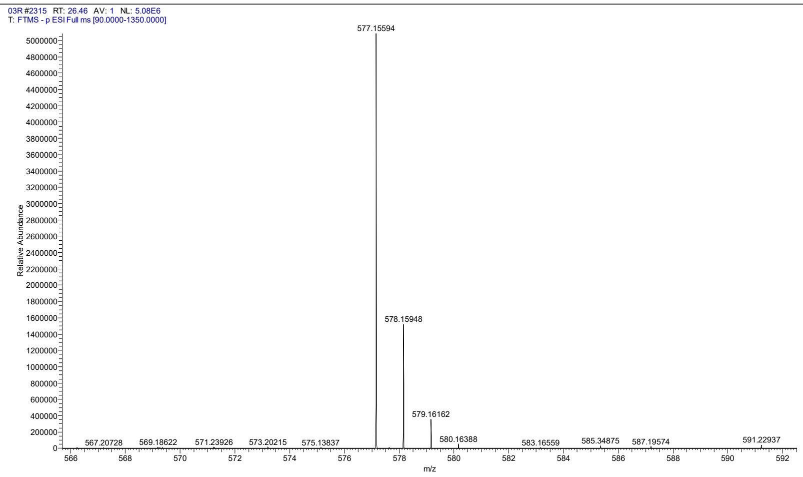

Forsythoside B 624.6

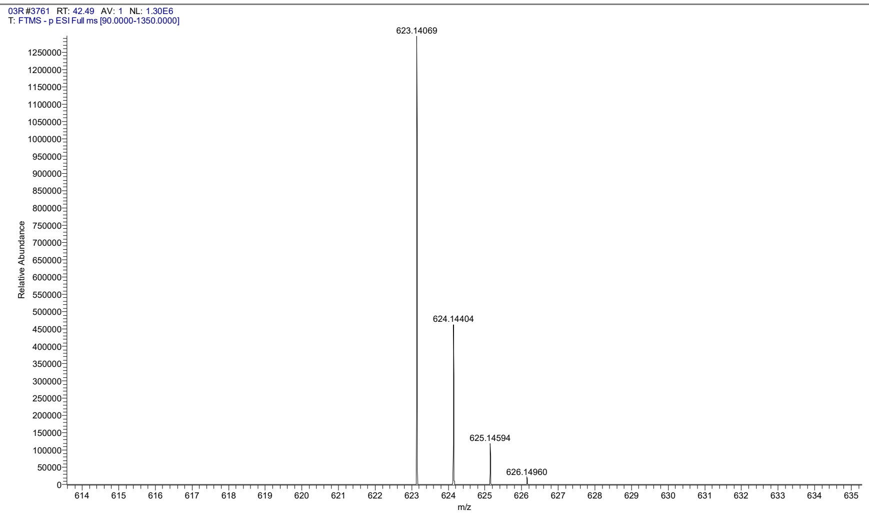

Heptadecane 240.47

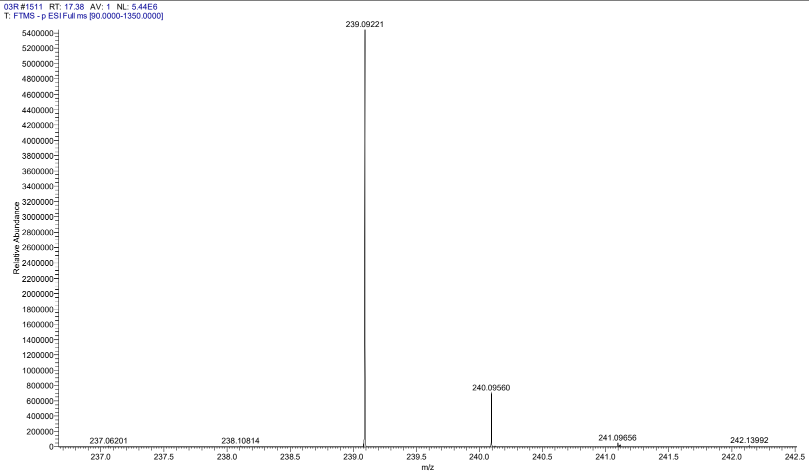

**Ladanein**	300.3	
**Leucosceptoside A**	610.6	

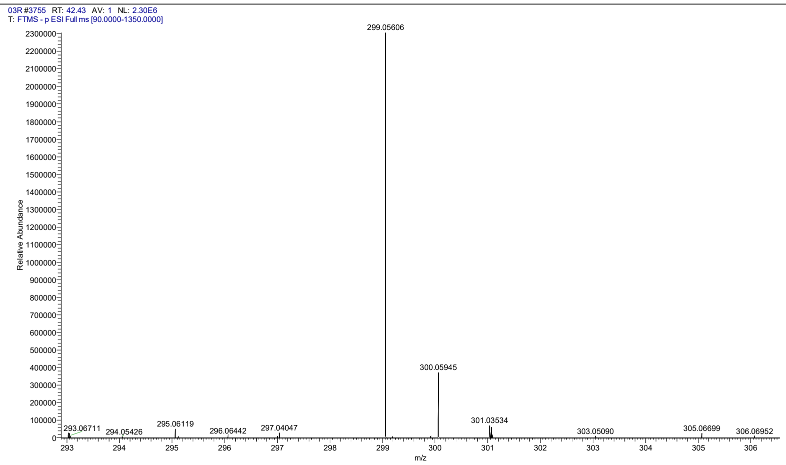

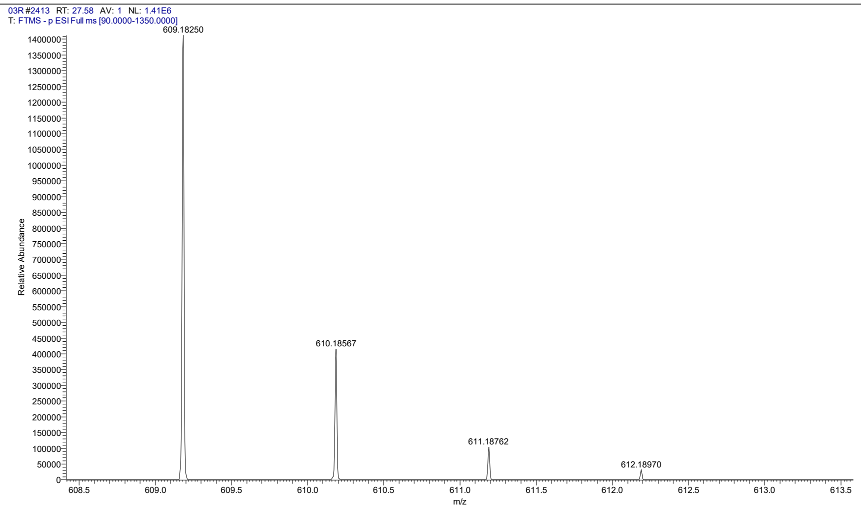

**Luteolin**	286.2	
**Luteolin 7-acetate**	344.3	

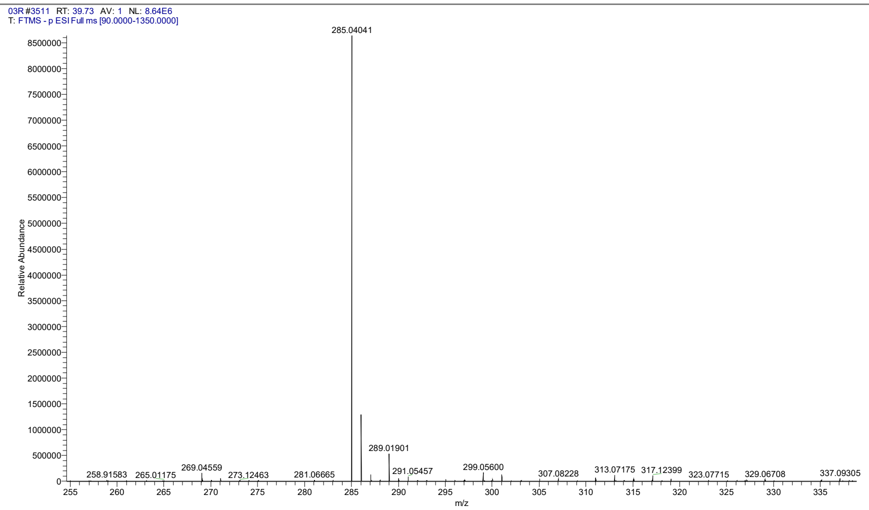

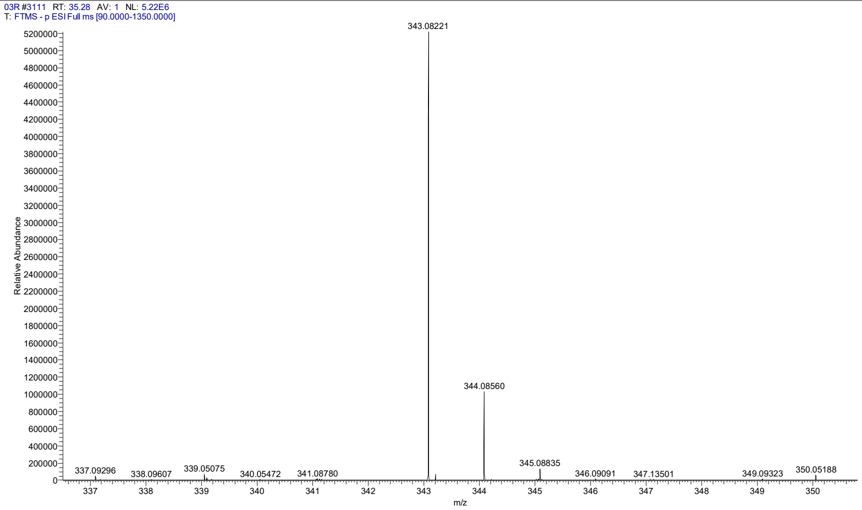

Luteolin 7-o-glucuronide

462.36

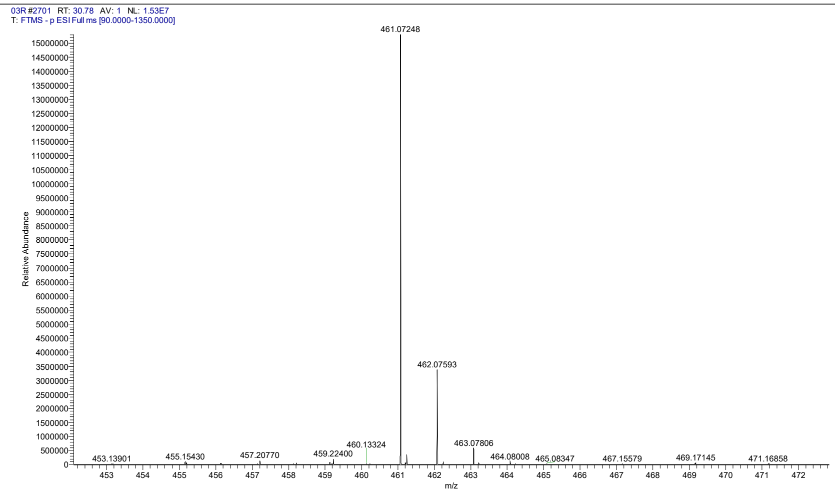

Luteolin 7-O-rutinoside

594.5

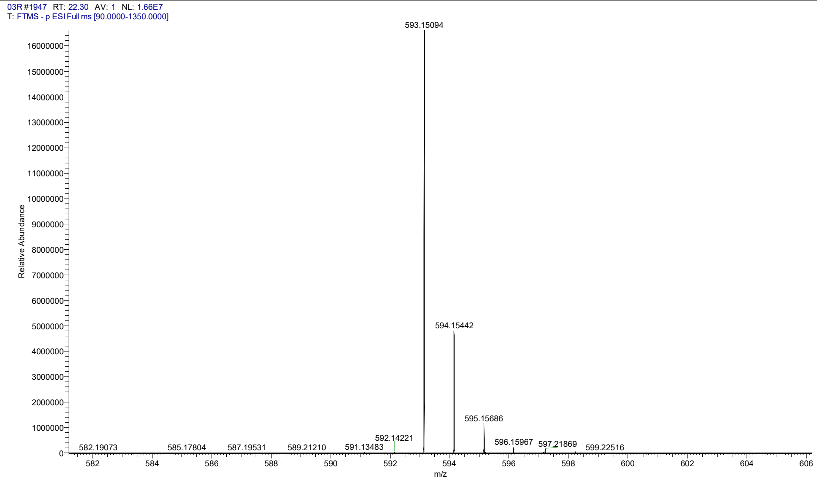

**Marrubenol**	336.47	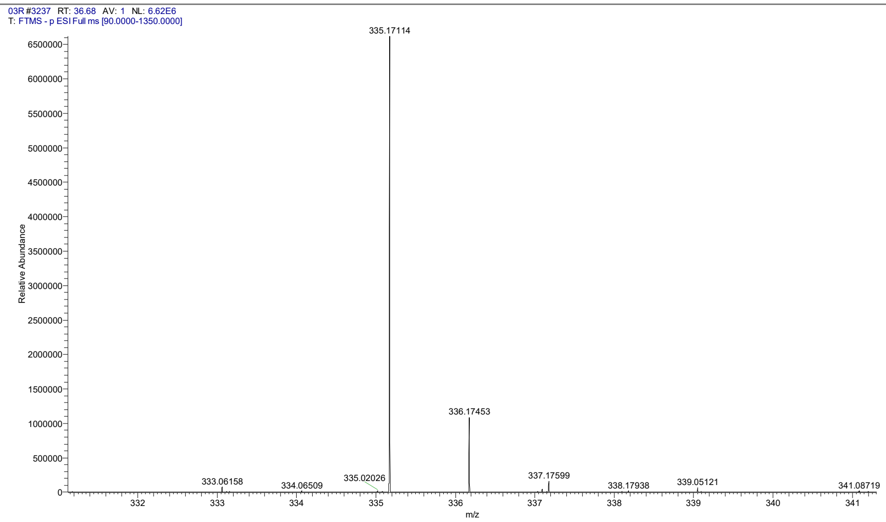
**Marrubiin**	536.7	
**Marruboside**	610.6	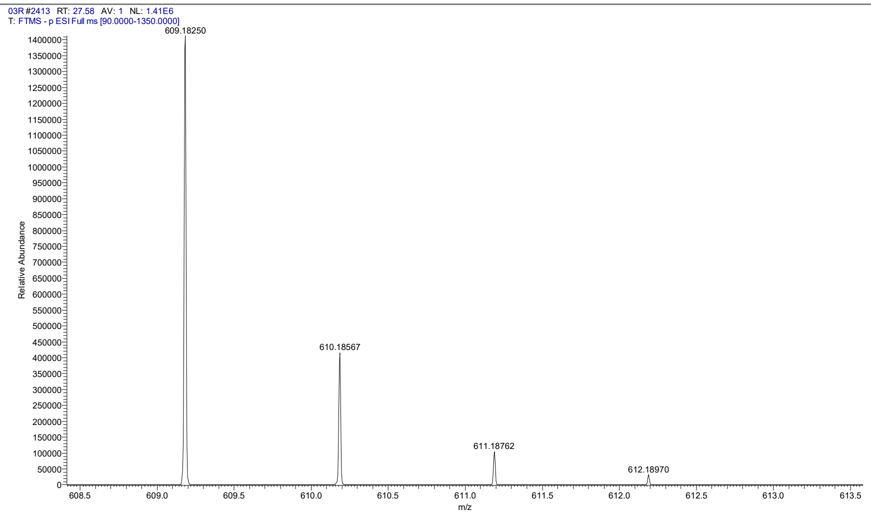

Marruliba-acetal 520.7

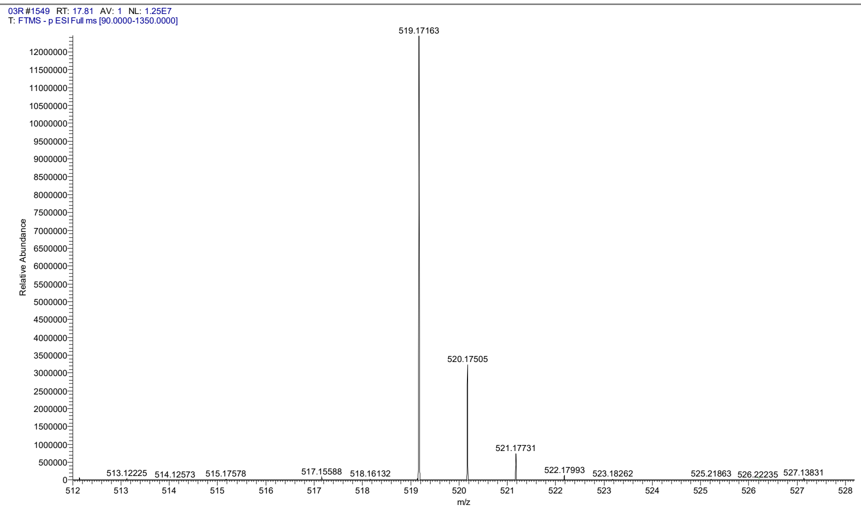

Oxacyclohexadecan-2-one 240.38

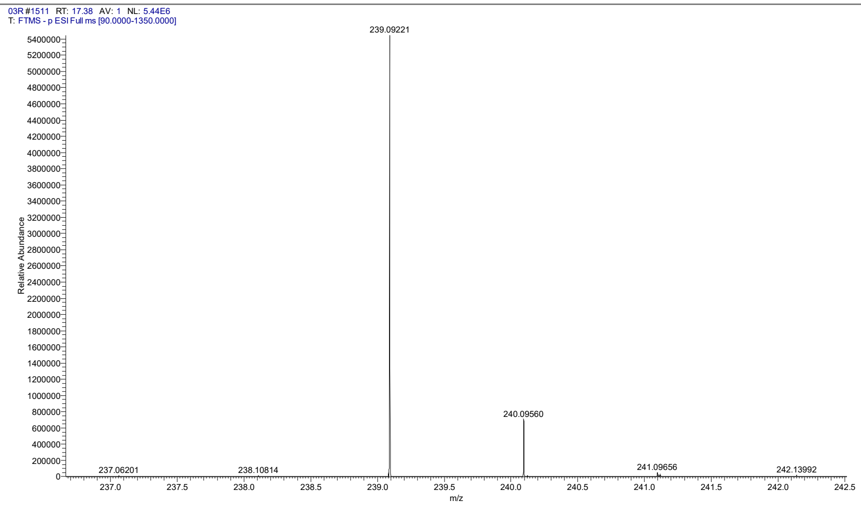

Polyodonine 520.7

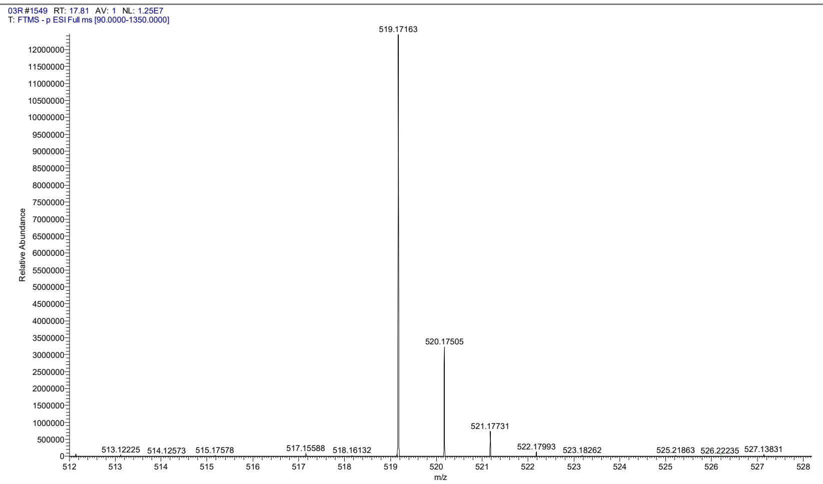

Preleosibirin 520.7

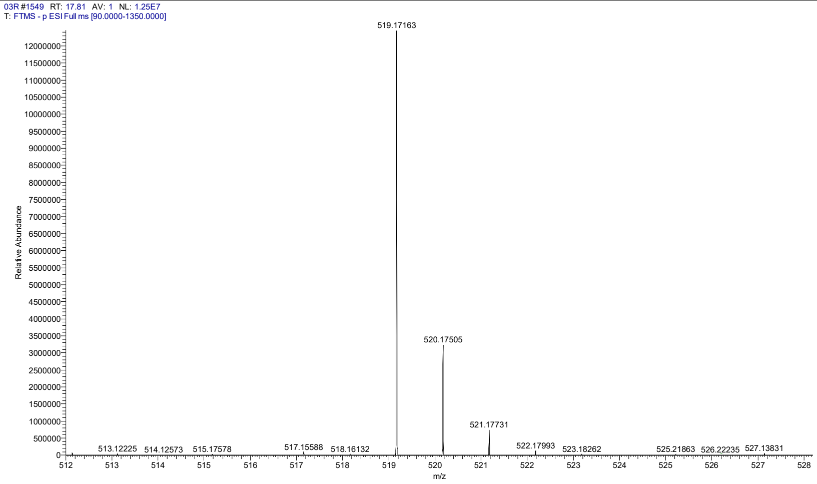

**Premarrubiin**	520.7	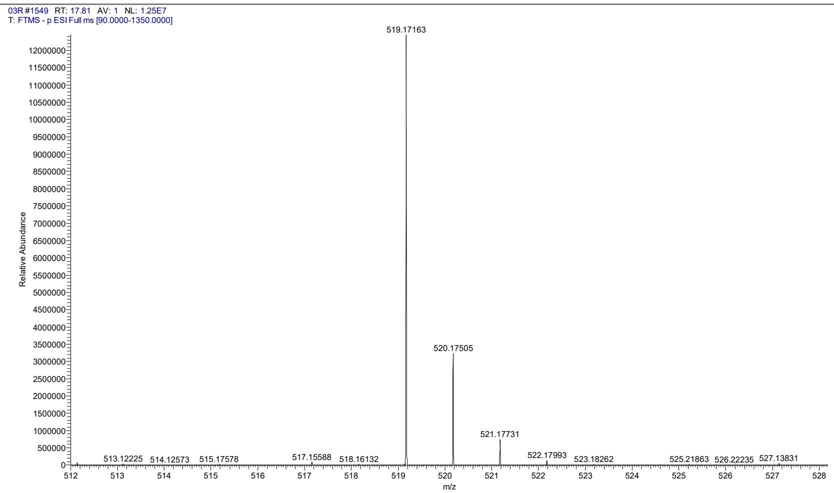
**Quercetin 3-O-rutinoside (Rutin)**	610.5	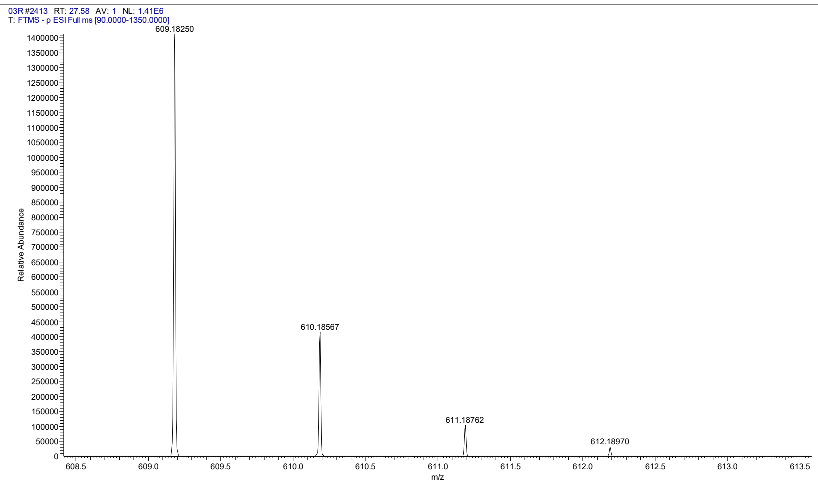

Rosmarinic acid 360.3

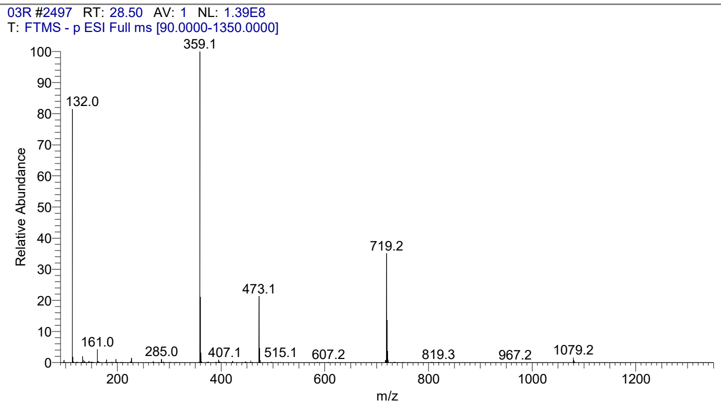

Samioside 610.6

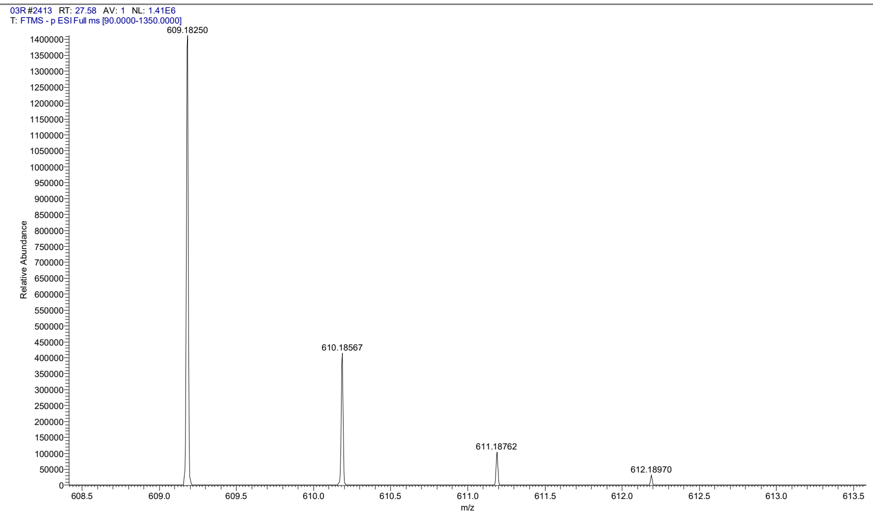

Syringic acid 198.2

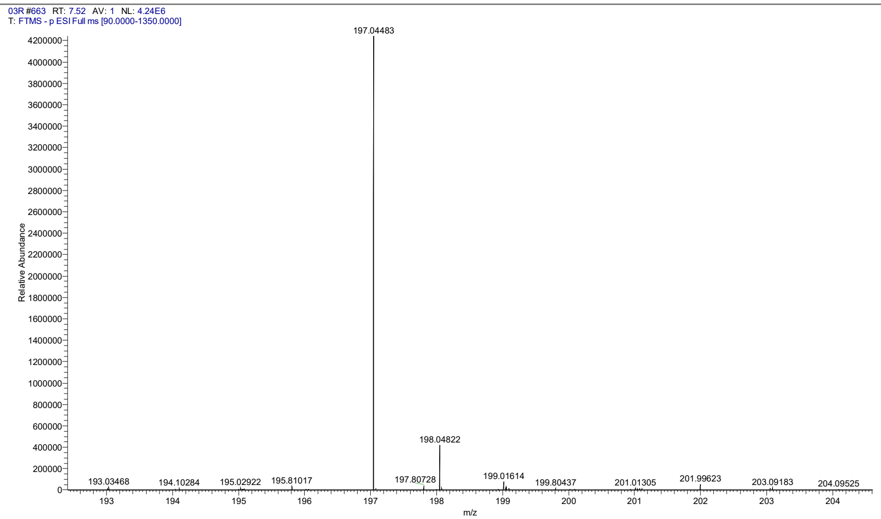

Verbascoside 624.59

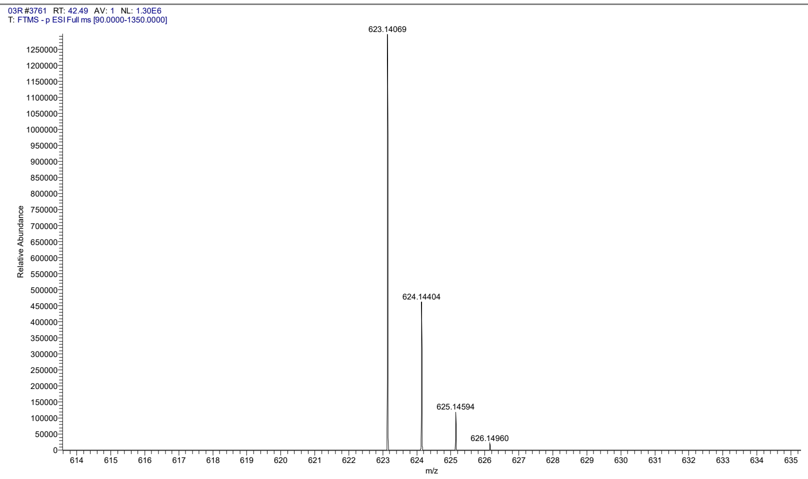

**Vicenin 2**	594.52	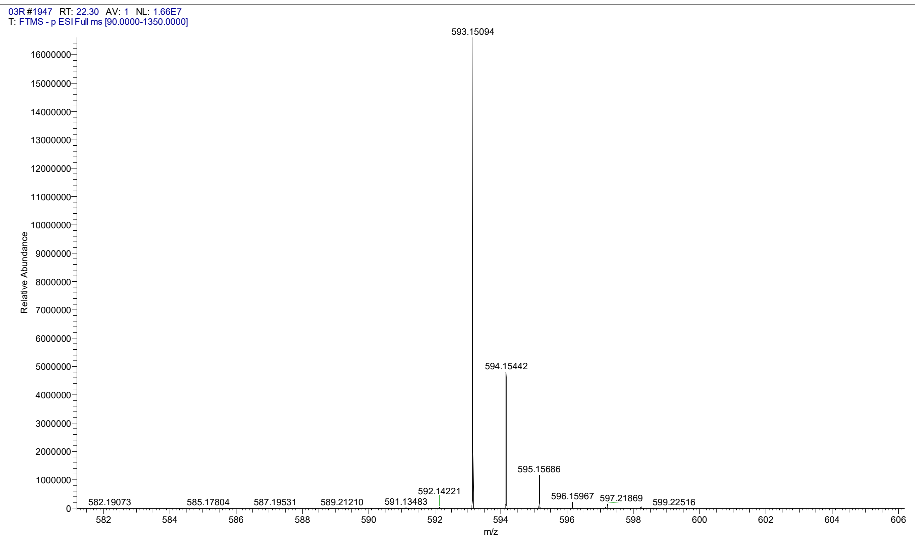

Organic solvent: Ethyl Acetate		
**Compound**	**Molecular weight**	**Ms spectrum**
Acacetin	284.3	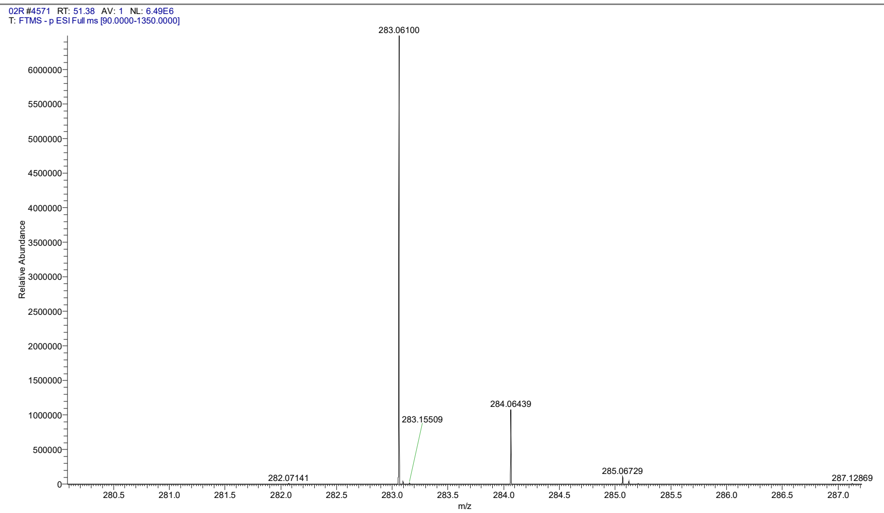
Apigenin 7-acetate	328.3	
Diosmetin	300.3	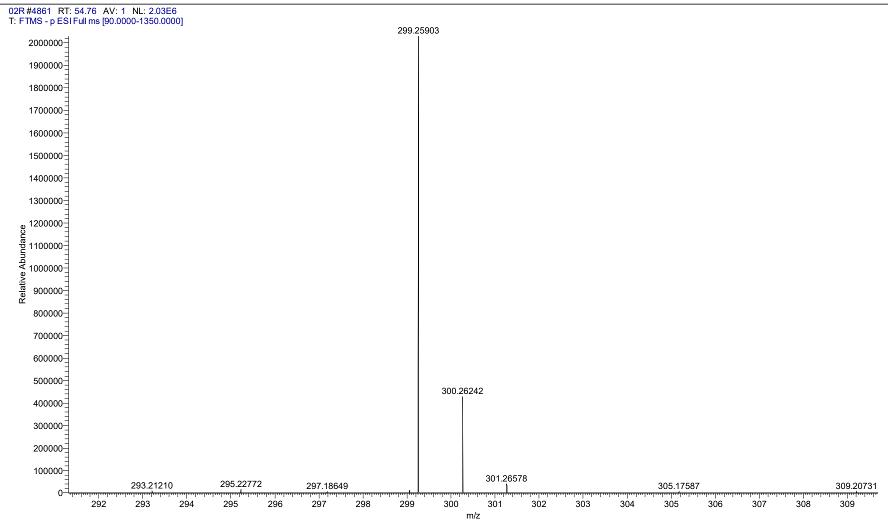

Diosmetin-7-O-glucoside	434.4	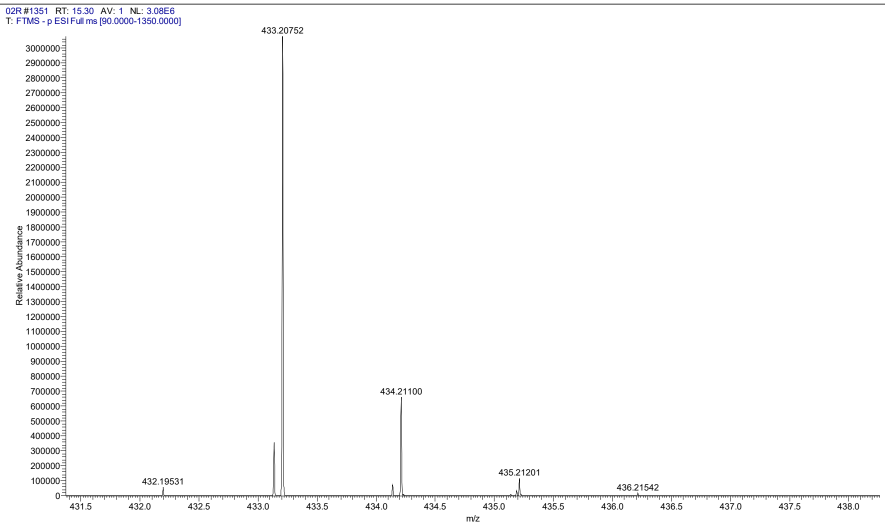
Docosane	310.60	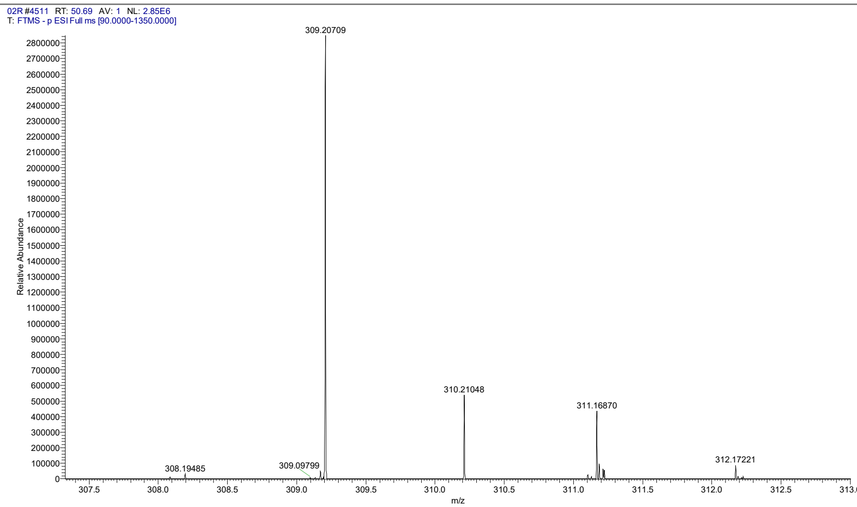

Galangin	270.2	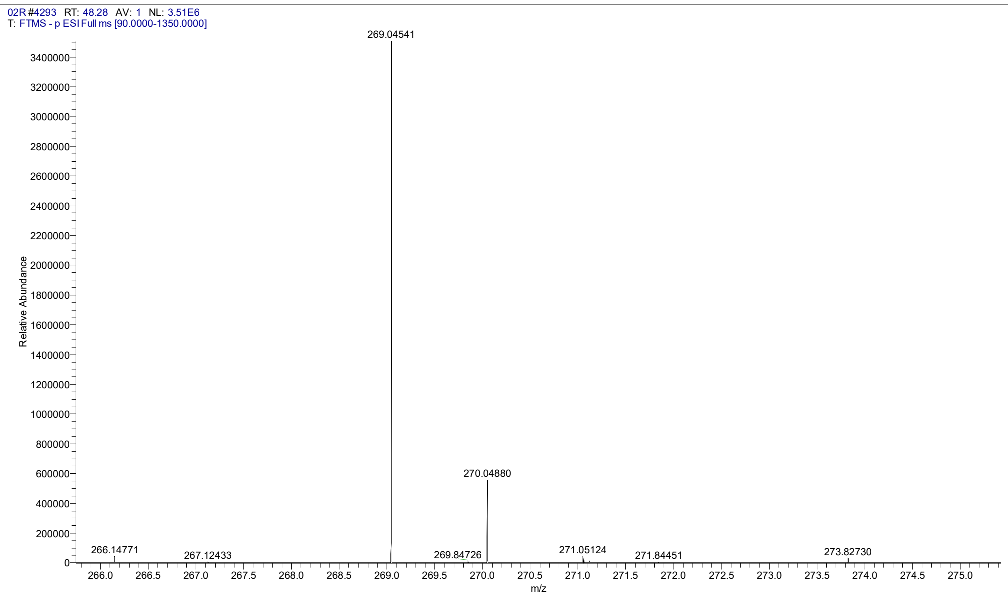
Ladanein	300.3	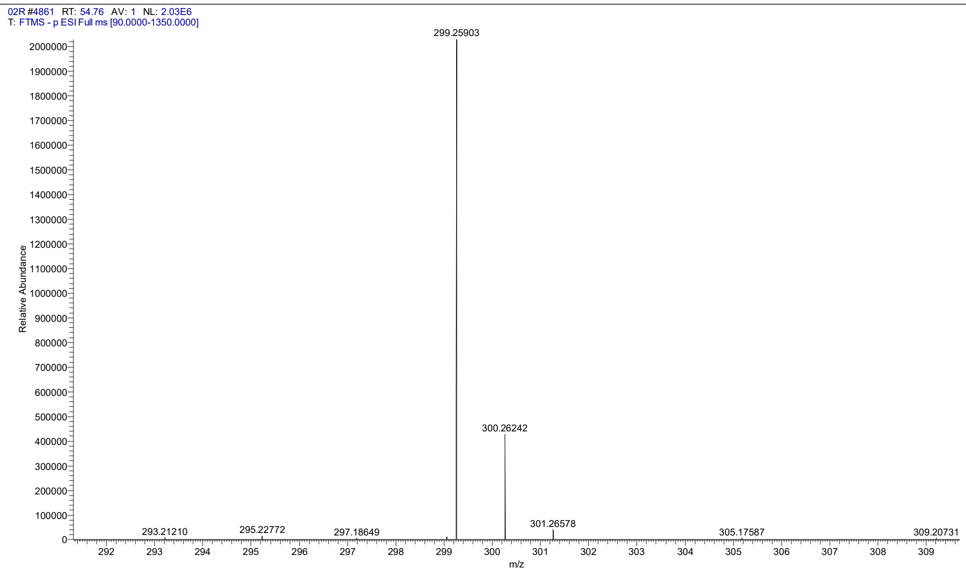

Marrubenol	332.5	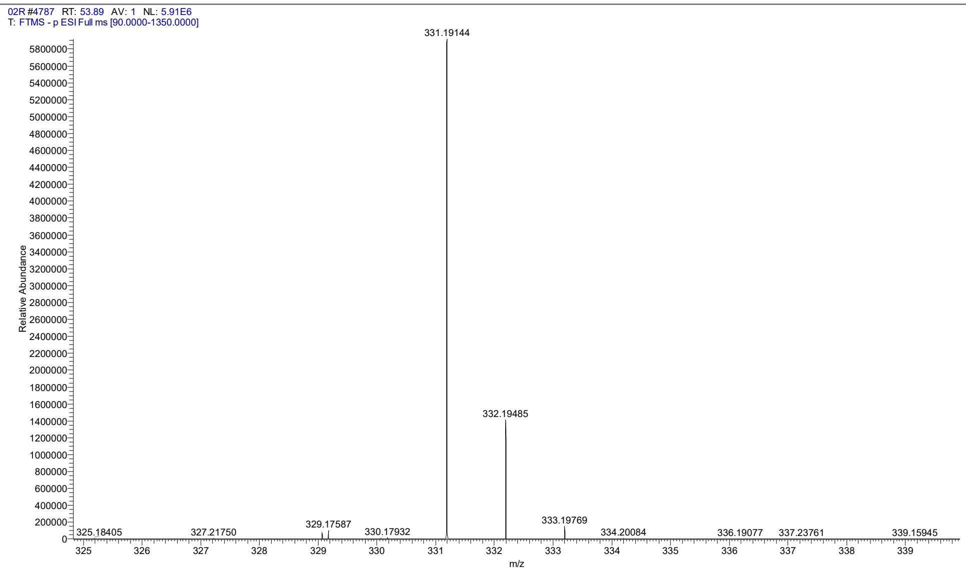
Marrubic acid	332.5	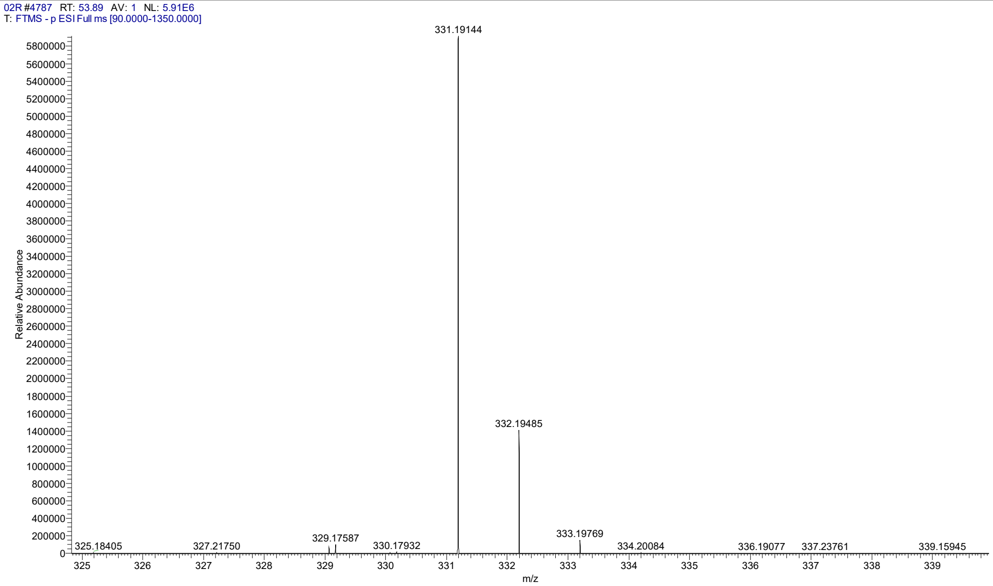

Marrubiin	332.44	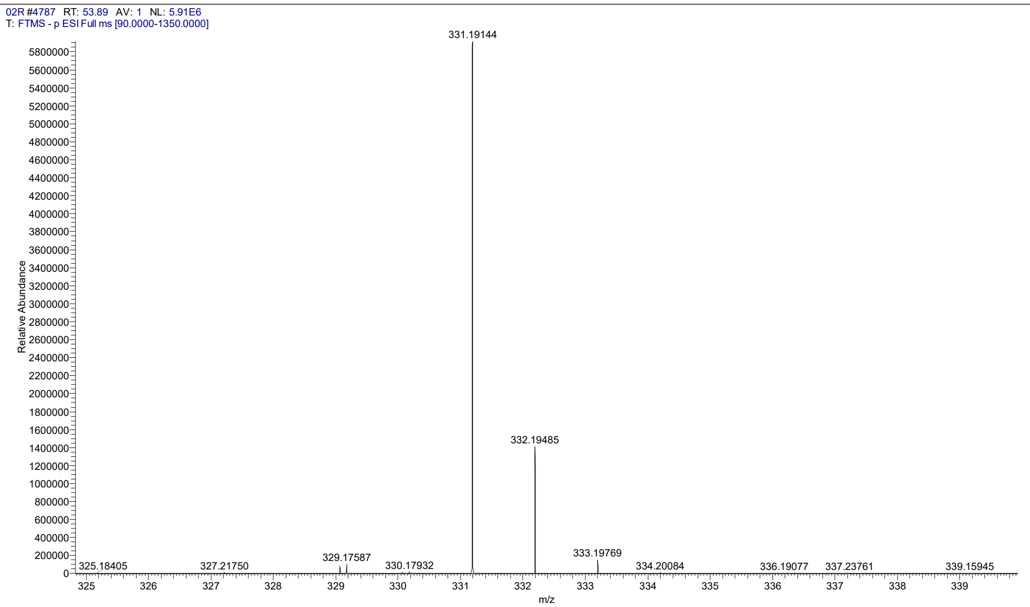
Methyl linoleate	294.47	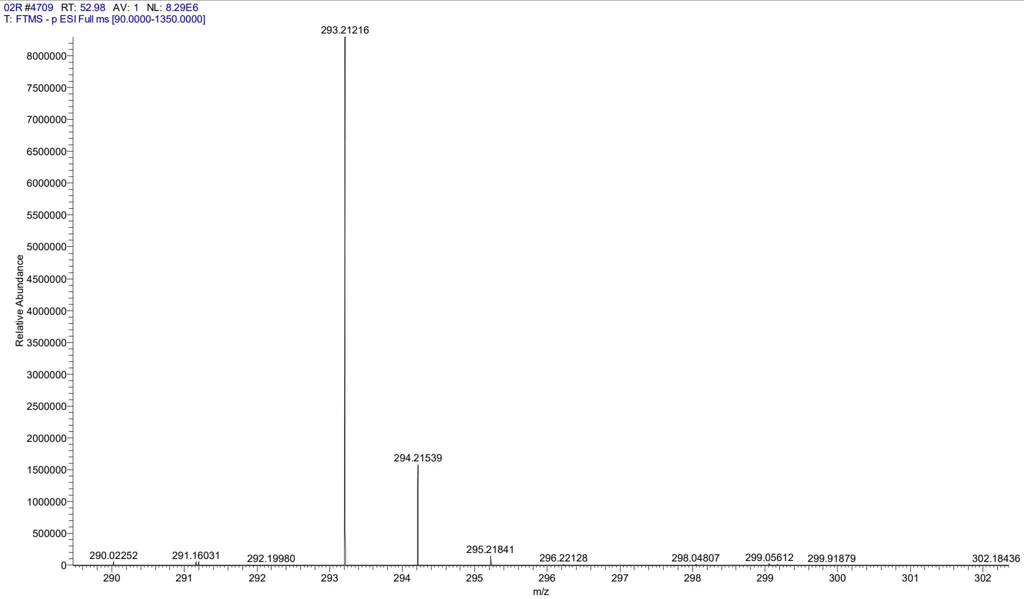

Peregrinin	332.5	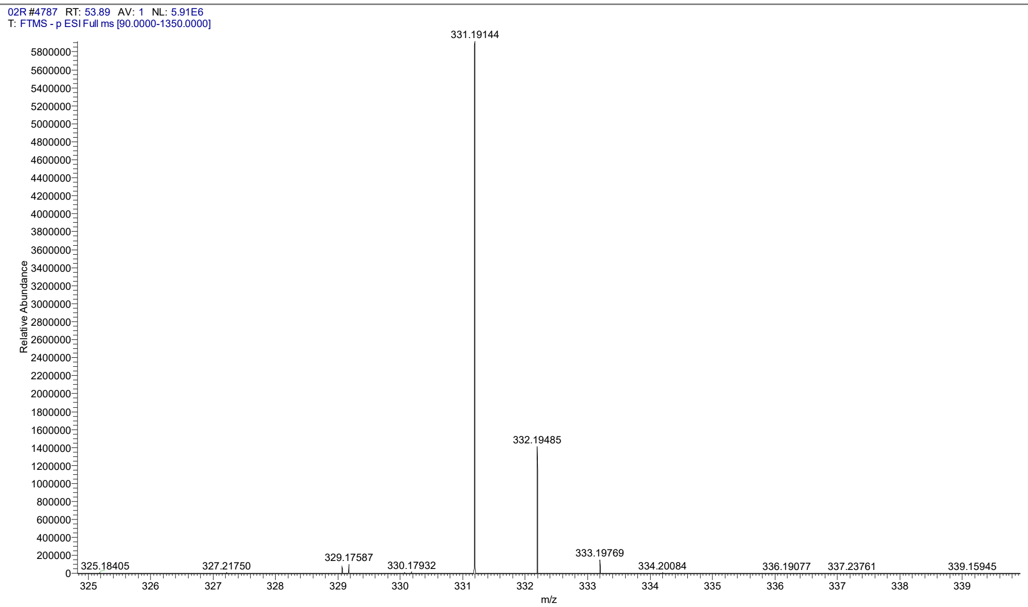
Peregrinol	332.5	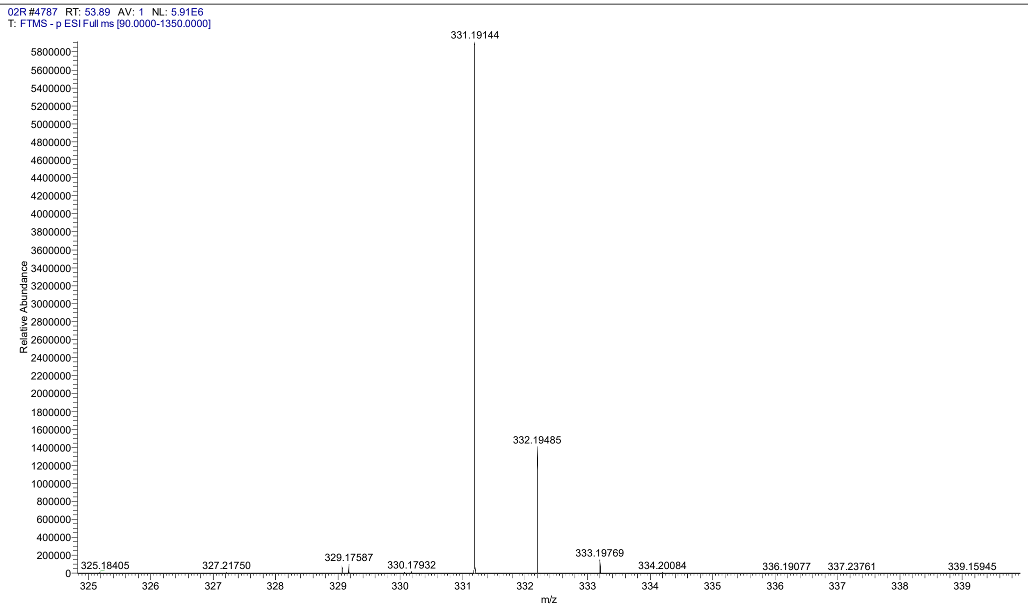

Premarrubiin	332.43	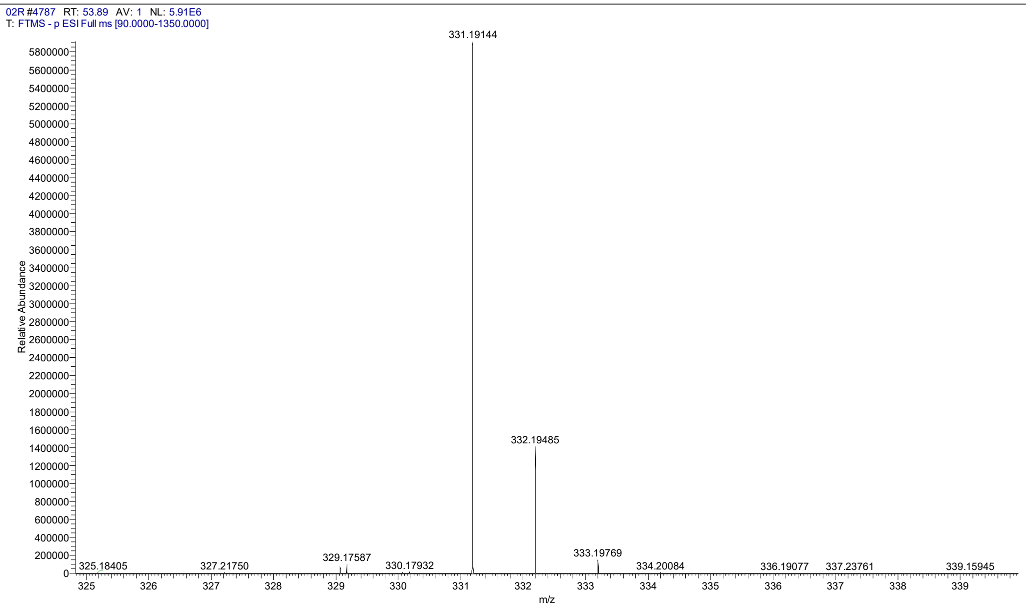
Rosmarinic acid	360.31	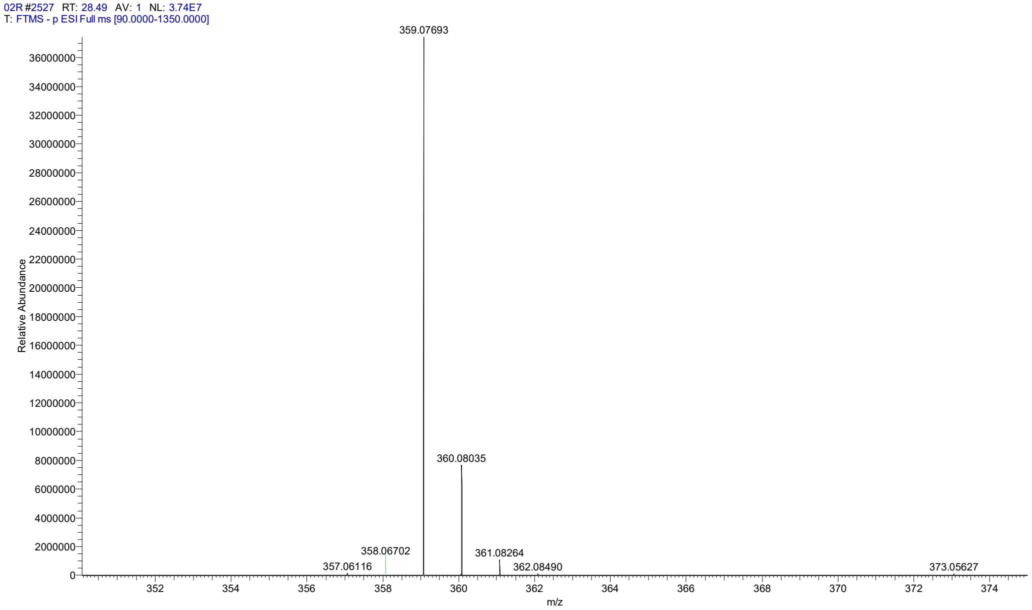

Stearyl alcohol 270.49

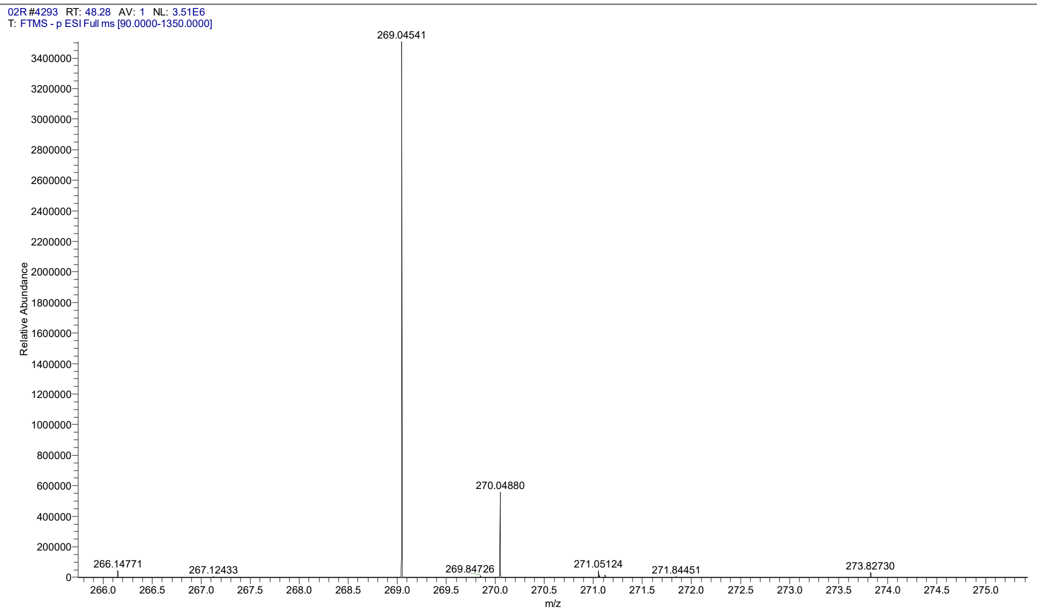

Vulgarol 332.5

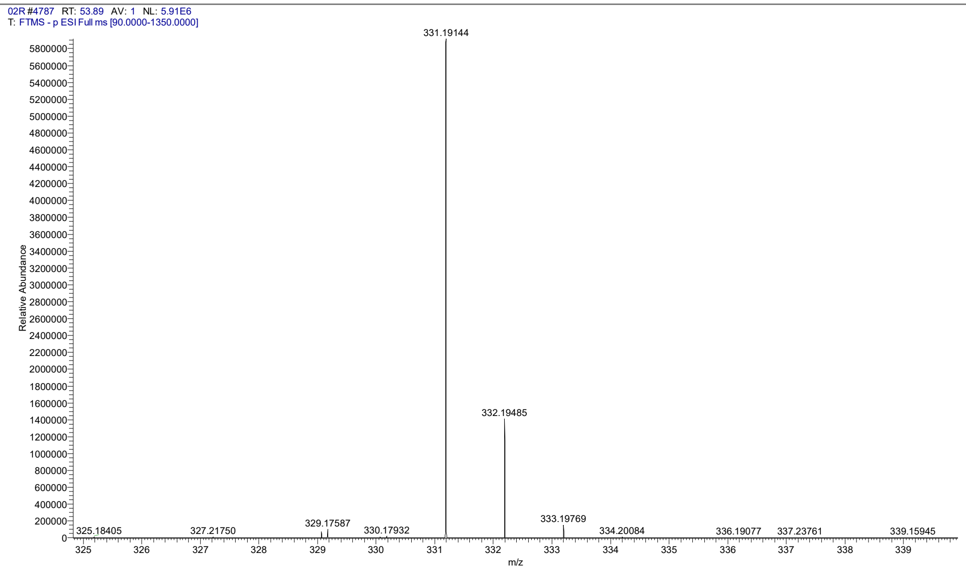

Organic solvent: Hexane

Compound	Molecular weight	Ms spectrum
**Chlorogenic acid**	354.3	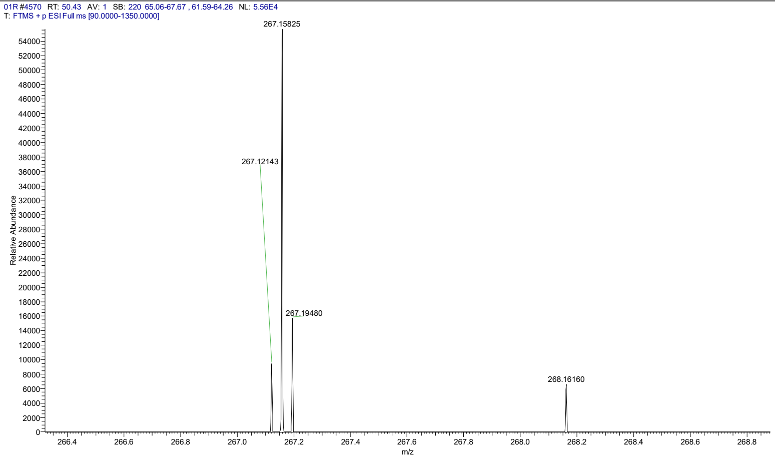
**Caffeoylmalic acid**	266.2	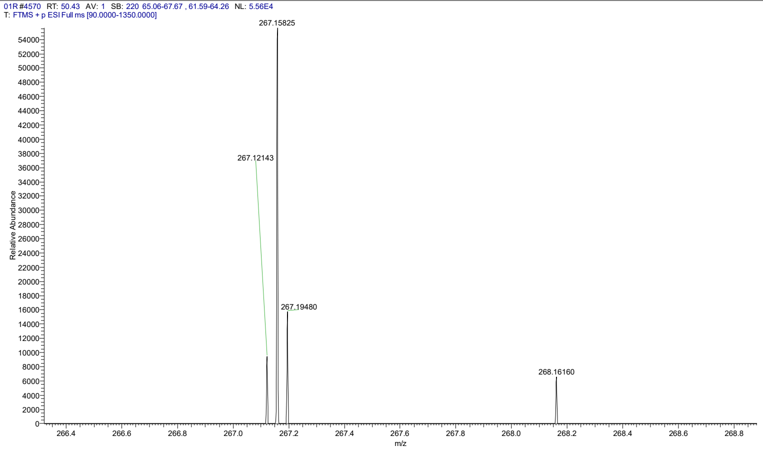

**Aesculin**	340.3	
**Ladanein**	314.29	

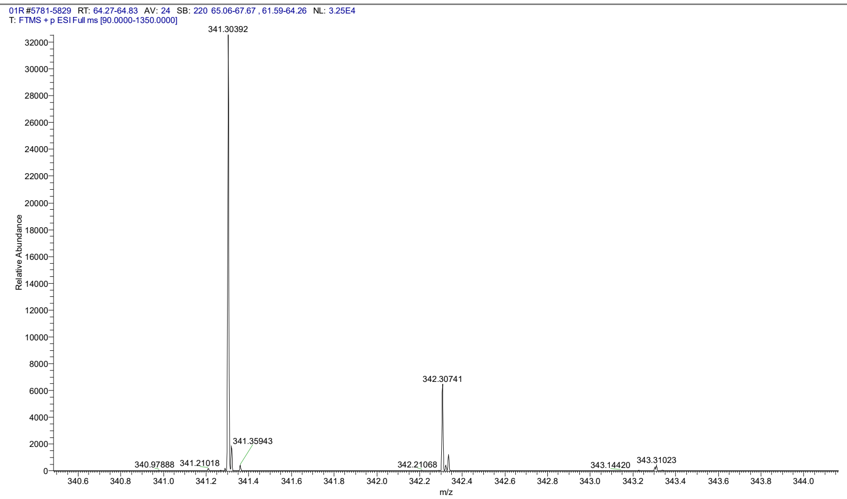

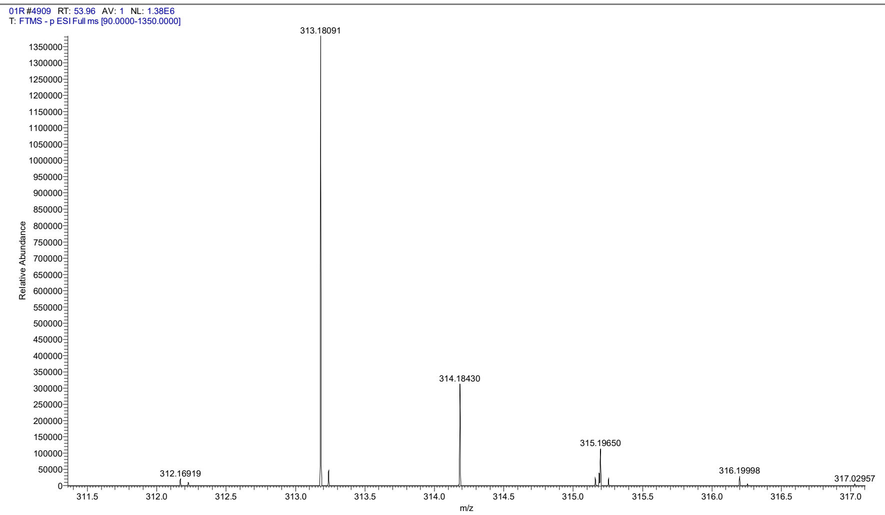

**Methyl linoleate**	294.47	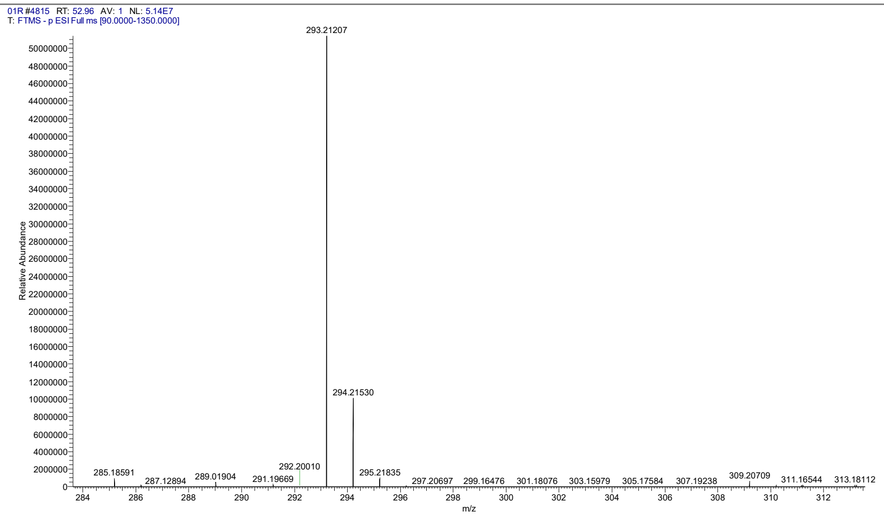
**Octadeca-2,4,6-trienoic acid**	278.43	
**Premarrubiin**	332.43	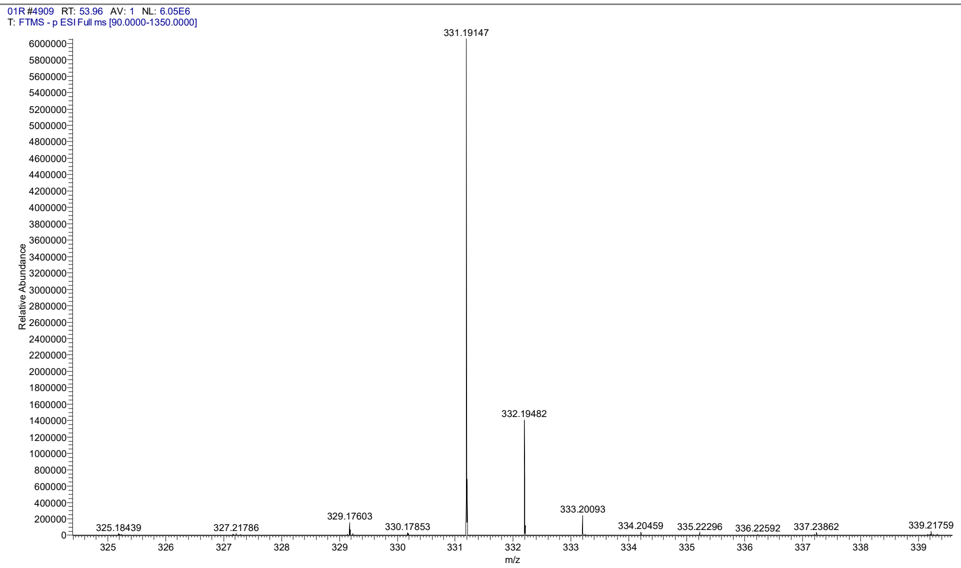

**Rosmarinic acid**	360.31	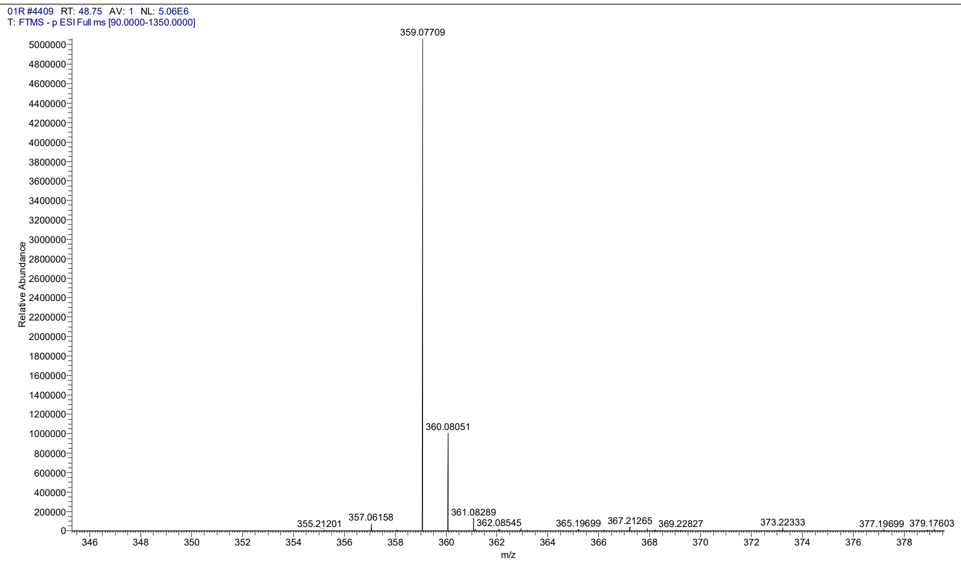
**cis-Piperitone oxide**	166.1	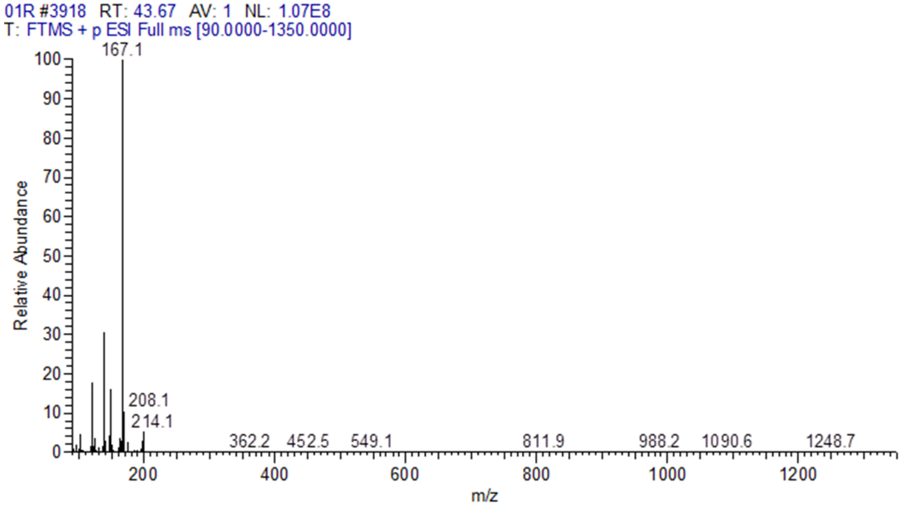

Aqueous extract		
**Compound**	Molecular weight	Ms spectrum
**Eugenol**	164.20	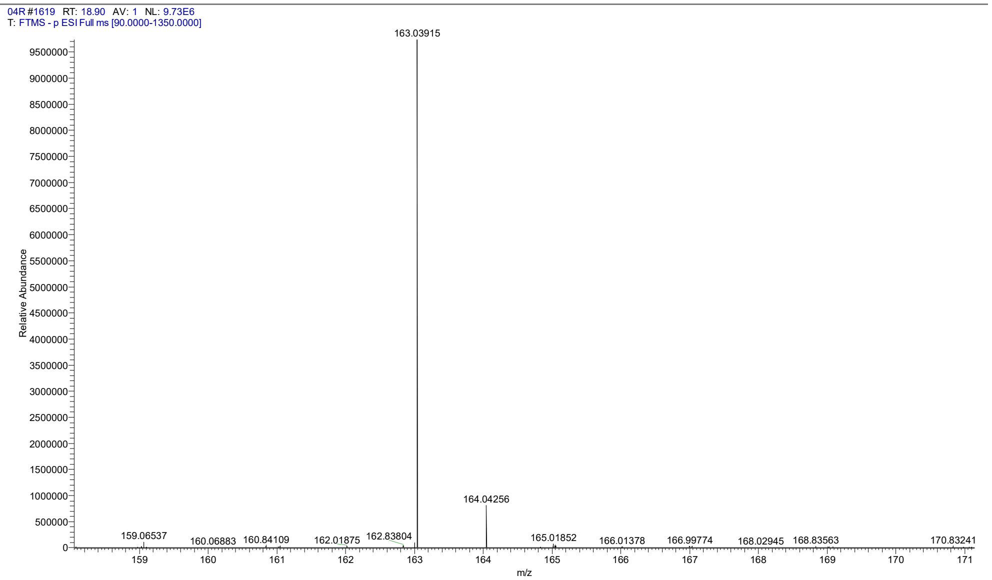

**Para-coumaric acid**	164.16	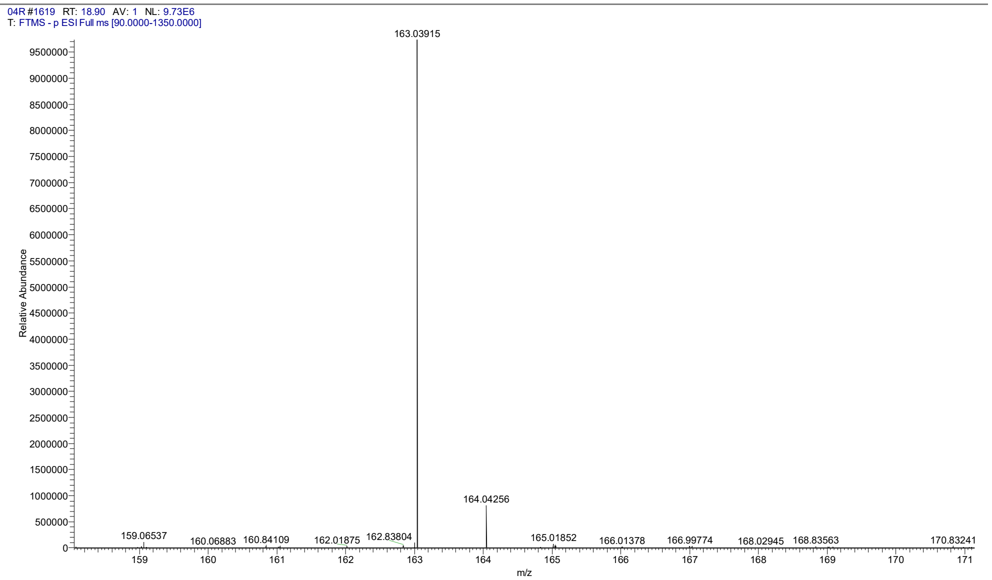
**Stigmast-5-en-3-ol**	414.71	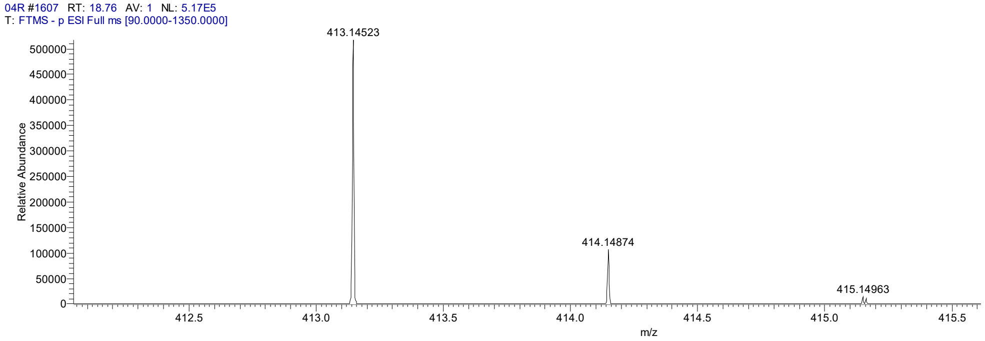

**β-sitosterol**	414.71	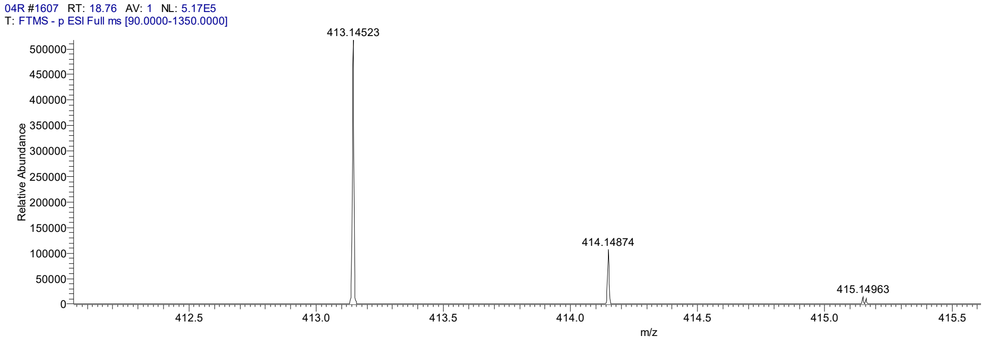
**Oxacyclohexadecan-2-one**	240.38	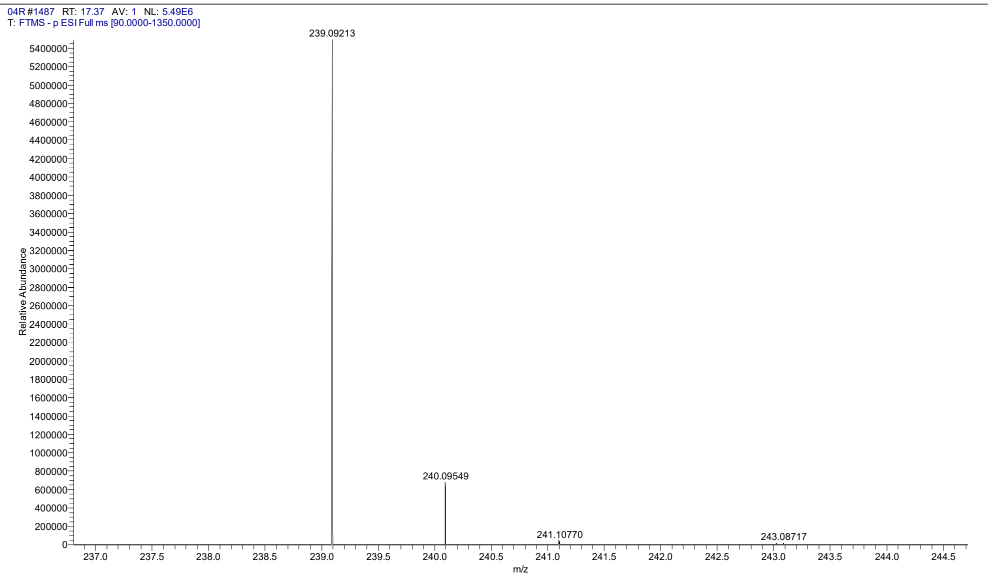

**Hexadecane**	226.45	
**o-Coumaric acid**	164.2	

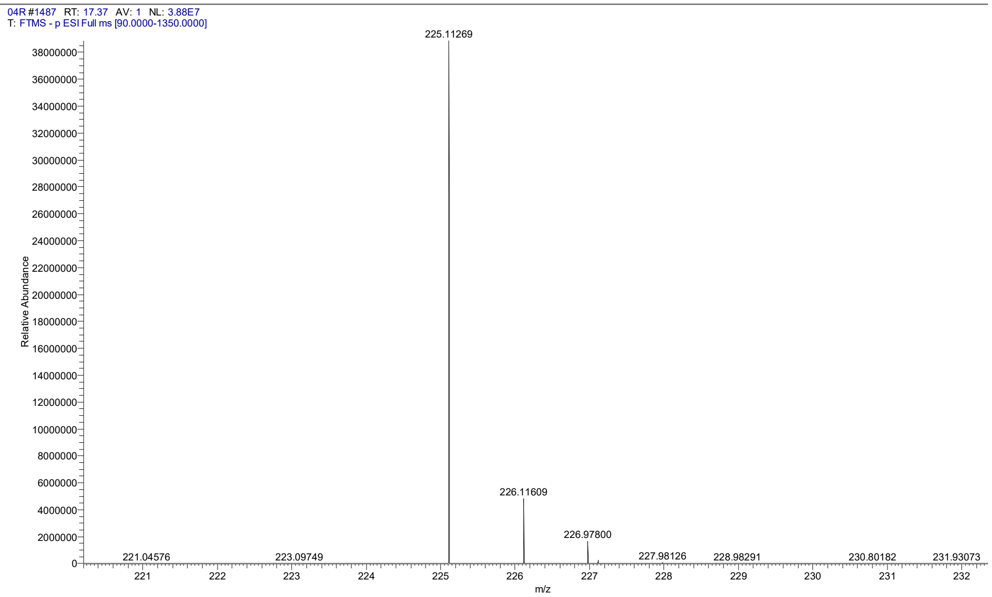

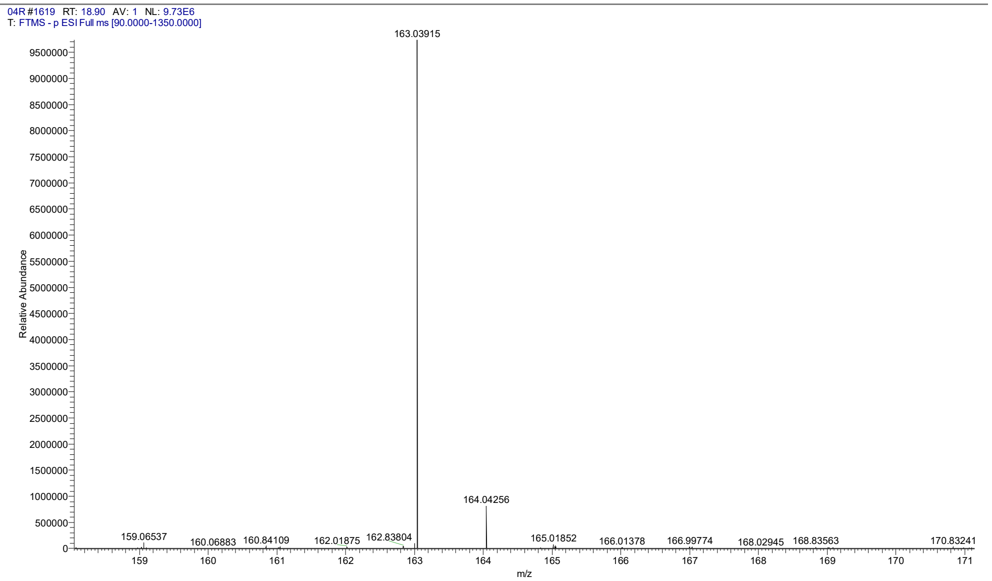

**p-Coumaric acid**	164.2	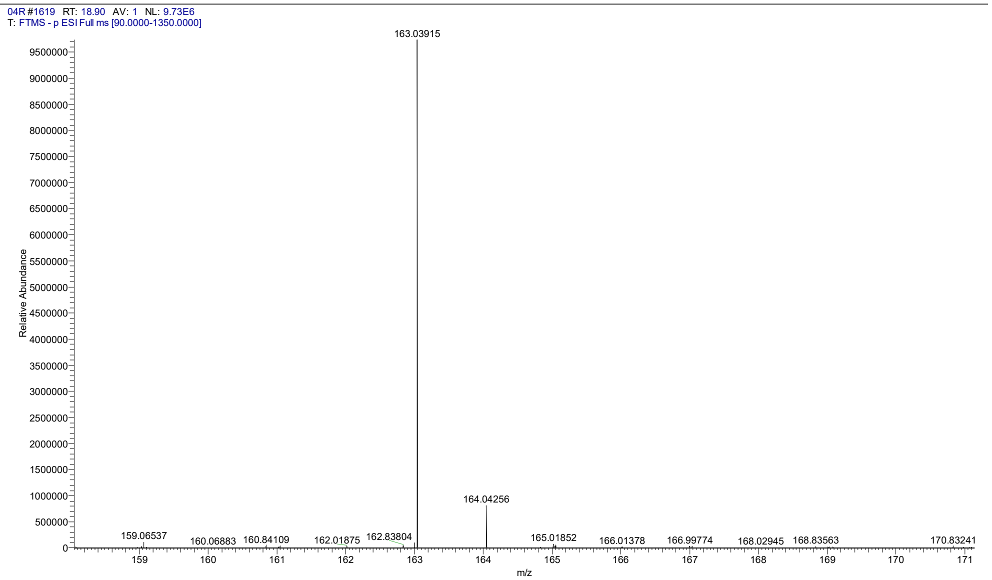
**Apigenin 7-acetate**	328.3	
