# Supplementary material for: Phytochemical Profiling and Structure-Based Computational Characterization of Marrubium vulgare L. Compounds as Hsp90 Modulators
Source: Int J Mol Sci. 2025 Dec 17;26(24):12150. doi: 10.3390/ijms262412150 (PMC12733462; doi:10.3390/ijms262412150)
Supplement: Supplementary file 1 [file ijms-26-12150-s001.zip › Table S6_SwissADME- Chlorogenic acid and Forsytoside B.pdf]

Table 6. SwissADME analysis of top hit compounds, including ADME parameters, drug-likeness, and medicinal chemistry as-sessment. A) Chlorogenic acid, B) Forsythoside B.

A)

Molecule 1: Chlorogenic Acid

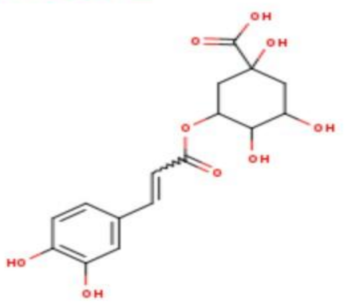

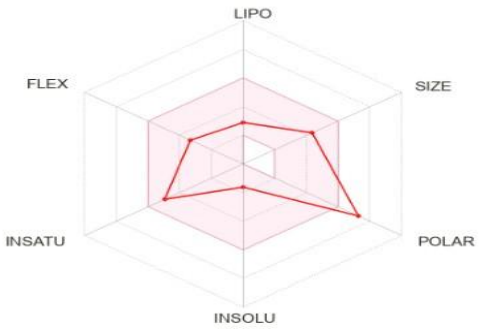

SMILES O=C(OC1CC(O)(CC(C1O)O)C(=O)O)C=Cc1ccc(c(c1)O)O

Physicochemical Properties	
Formula	C16H18O9
Molecular weight	354.31 g/mol
Num. heavy atoms	25
Num. arom. heavy atoms	6
Fraction Csp3	0.38
Num. rotatable bonds	5
Num. H-bond acceptors	9
Num. H-bond donors	6
Molar Refractivity	83.50
TPSA	164.75 Å²

Lipophilicity	
Log $P_{o/w}$ (iLOGP)	0.96
Log $P_{o/w}$ (XLOGP3)	-0.42
Log $P_{o/w}$ (WLOGP)	-0.75
Log $P_{o/w}$ (MLOGP)	-1.05
Log $P_{o/w}$ (SILICOS-IT)	-0.61
Consensus Log $P_{o/w}$	-0.38

Water Solubility	
Log *S* (ESOL)	-1.62
Solubility	8.50e+00 mg/ml ; 2.40e-02 mol/l
Class	Very soluble
Log *S* (Ali)	-2.58
Solubility	9.42e-01 mg/ml ; 2.66e-03 mol/l
Class	Soluble
Log *S* (SILICOS-IT)	0.40
Solubility	8.94e+02 mg/ml ; 2.52e+00 mol/l
Class	Soluble

Pharmacokinetics	
GI absorption	Low
BBB permeant	No
P-gp substrate	No
CYP1A2 inhibitor	No
CYP2C19 inhibitor	No
CYP2C9 inhibitor	No
CYP2D6 inhibitor	No
CYP3A4 inhibitor	No
Log K_p (skin permeation)	-8.76 cm/s

Druglikeness	
Lipinski	Yes; 1 violation: NHorOH>5
Ghose	No; 1 violation: WLOGP<-0.4
Veber	No; 1 violation: TPSA>140
Egan	No; 1 violation: TPSA>131.6
Muegge	No; 2 violations: TPSA>150, H-don>5
Bioavailability Score	0.11

Medicinal Chemistry	
PAINS	1 alert: catechol_A
Brenk	2 alerts: catechol, michael_acceptor_1
Leadlikeness	No; 1 violation: MW>350
Synthetic accessibility	4.16

B)

Molecule 2: Forsythoside B

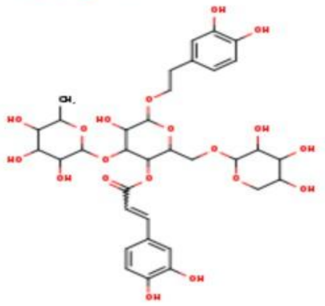

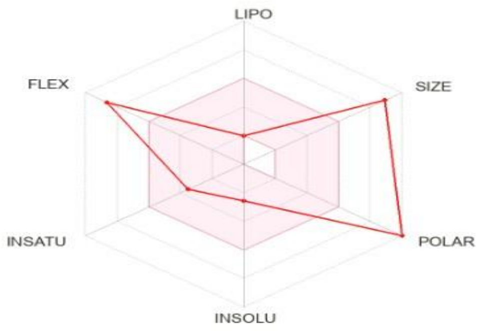

SMILES O=C(OC1C(COC2OCC(C(C2O)O)O)OC(C(C1OC1OC(C)C(C(C1O)O)O)O)OCCc1ccc(c(c1)O)O)C=Cc1ccc(c(c1)O)O

Physicochemical Properties	
Formula	C34H44O19
Molecular weight	756.70 g/mol
Num. heavy atoms	53
Num. arom. heavy atoms	12
Fraction Csp3	0.56
Num. rotatable bonds	13
Num. H-bond acceptors	19
Num. H-bond donors	11
Molar Refractivity	174.84
TPSA	304.21 Å²

Lipophilicity	
Log $P_{o/w}$ (iLOGP)	1.63
Log $P_{o/w}$ (XLOGP3)	-2.03
Log $P_{o/w}$ (WLOGP)	-2.66
Log $P_{o/w}$ (MLOGP)	-3.93
Log $P_{o/w}$ (SILICOS-IT)	-2.74
Consensus Log $P_{o/w}$	-1.95

Water Solubility	
Log *S* (ESOL)	-2.56
Solubility	2.07e+00 mg/ml ; 2.74e-03 mol/l
Class	Soluble
Log *S* (Ali)	-3.83
Solubility	1.11e-01 mg/ml ; 1.47e-04 mol/l
Class	Soluble
Log *S* (SILICOS-IT)	1.18
Solubility	1.14e+04 mg/ml ; 1.51e+01 mol/l
Class	Soluble

Pharmacokinetics	
GI absorption	Low
BBB permeant	No
P-gp substrate	No
CYP1A2 inhibitor	No
CYP2C19 inhibitor	No
CYP2C9 inhibitor	No
CYP2D6 inhibitor	No
CYP3A4 inhibitor	No
Log K_p (skin permeation)	-12.36 cm/s

Druglikeness	
Lipinski	No; 3 violations: MW>500, NorO>10, NHorOH>5
Ghose	No; 4 violations: MW>480, WLOGP<-0.4, MR>130, #atoms>70
Veber	No; 2 violations: Rotors>10, TPSA>140
Egan	No; 1 violation: TPSA>131.6
Muegge	No; 5 violations: MW>600, XLOGP3<-2, TPSA>150, H-acc>10, H-don>5
Bioavailability Score	0.17

Medicinal Chemistry	
PAINS	1 alert: catechol_A
Brenk	2 alerts: catechol, michael_acceptor_1
Leadlikeness	No; 2 violations: MW>350, Rotors>7
Synthetic accessibility	7.28
